# “It has tentacles into every single aspect of me” a qualitative evidence synthesis of the lived experiences and perceptions of ADHD youth

**DOI:** 10.1007/s00787-025-02955-8

**Published:** 2026-02-25

**Authors:** Jessie Tierney, Ann-Marie Morrissey, Dimitrios Adamis, Margo Wrigley, Katie Robinson

**Affiliations:** 1 Health Research Institute, School of Allied Health, Faculty of Education and Health Sciences, University of Limerick, Limerick, Ireland; 2 Ageing Research Centre, Health Research Institute, School of Allied Health, Faculty of Education and Health Sciences, University of Limerick, Limerick, Ireland; 3 Sligo Mental Health Services Adult ADHD Clinic, Sligo, Ireland; 4 Department of Psychiatry, University of Limerick, Limerick, Ireland; 5 Health Service Executive, HSE National Clinical Programme for ADHD in Adults, Dublin, Dublin 8 Ireland

**Keywords:** ADHD, Youth, Lived experience, Qualitative, Synthesis

## Abstract

Attention Deficit Hyperactivity Disorder (ADHD) is a neurodevelopmental disorder affecting up to 5.9% of youth. Characterised by inattention, hyperactivity, and impulsivity, it affects various aspects of functioning and is often accompanied with co-occurring diagnoses. While ADHD poses significant challenges, including academic and occupational impairments, it can also confer strengths like creativity and hyperfocus. Successfully navigating the transition to adulthood is crucial for improving long-term health and vocational outcomes for ADHD youth. The aim of this review is to identify and synthesise existing qualitative literature on the experiences of ADHD youth across life domains. A qualitative evidence synthesis, utilising meta-ethnographic methods, with purposive sampling was conducted. Thirty studies, across 11 countries, reporting the experiences of 805 ADHD youth were analysed. Themes generated were: (1) Navigating identity and authenticity (2) Diagnosis disclosure dilemma, (3) New ADHD manager reporting for duty, (4) The power of others, (5) Diverse healthcare journeys, (6) Impact on engagement in everyday activities, (7) Turning insight to action. Findings illustrate the wide-ranging impacts of ADHD across life domains, particularly education, employment and leisure. Transitioning to adulthood, youth assume responsibility for managing their ADHD, with differing levels of readiness. While social connections offer vital support, many struggle to form or maintain these relationships. The health service journeys of youth vary, in accessibility, continuity of care and patient-clinician relationship. Effective support requires strengths-based, collaborative, and continuity-focused approaches to education, employment, and healthcare transitions.

**Supplementary Information:**

The online version contains supplementary material available at 10.1007/s00787-025-02955-8.

## Background

Attention Deficit Hyperactivity Disorder (ADHD) is a neurodevelopmental disorder signified by differences in brain processes affecting personal, social, academic, and/or overall occupational functioning [1]. The core features of ADHD - inattention and hyperactivity-impulsivity - were previously considered as fixed subtypes but are now recognised as dynamic presentations [2]. Furthermore, ADHD and its primary features are regarded as dimensional rather than categorical, meaning that an individual is recognised as having the disorder only when these characteristics produce a moderate to severe impact across two or more areas of functioning [3]. In addition to key features, individuals may experience challenges with problem solving, memory, and emotional regulation [4]. However, many with lived experience also highlight positive features such as high energy levels, spontaneity, hyperfocus [5], ‘outside the box’ thinking [6], creativity, and increased empathy [7].

A significant portion of individuals with childhood ADHD continue to experience symptoms into adulthood, known as persistent ADHD [8–10]. Globally, an estimated 2.58% of adults live with persistent ADHD, with the highest prevalence among youth [11]. According to Eurostat, the statistical office of the European Commission, *youth* refers to individuals aged 15-to-29-years-old, encompassing the transition to adulthood marked by completion of compulsory education, beginning career development, and attaining financial responsibilities [12–14].

Although there is significant variation in the level of functional impairment, severity of symptoms, and development of co-occurring psychological diagnoses [15, 16], ADHD is associated with academic impairment, negative occupational functioning, low self-esteem and peer rejection [4]. By approximately 14 years of age, ADHD students are on average 2.5 years behind in reading and 4.5 years in writing than non-ADHD peers [17], in high school they are significantly more likely to repeat a grade [18], have higher rates of absenteeism and are up to 2.7 times more likely to drop out before graduating [19]. Those with higher levels of symptomatology at the outset of their higher education program have poorer long-term academic success [20]. ADHD college students also experience lower levels of social adjustment, self-reported social skills and self-esteem during college, than non-ADHD peers [21]. As youth enter the workforce, ADHD has an indirect and direct impact on job attainment, indirect as educational achievements may be negatively affected by attentional issues, and direct as they may impulsively quit, become restless and bored, and/or have difficulty with timekeeping [22]. When reflecting on their career path, ADHD adults report feelings of lost potential, despite an awareness of their high capability they still felt limited by their ADHD [23]. Despite this ADHD workers may not qualify for workplace supports or accommodations, as laws tend to be elusive when concerning ADHD in certain countries [24]. Additionally, as many as 89% of ADHD adults experience one or more co-occurring condition, such as anxiety, mood, substance use, personality disorders and sleep disturbance [25–29], and ADHD and Autism have high rates of co-diagnosis [30], further compounding the challenges individuals face in achieving educational and occupational outcomes.

Safe and effective pharmacological treatments for ADHD are available and fall broadly into two categories: stimulant and non-stimulant medications [4]. A review and meta-analysis of the effects of ADHD medication on functional outcomes found robust protective effects across multiple domains, including mood disorders, criminality, substance use disorders, suicidality, accidents and injury, and educational outcomes [31]. However, premature discontinuation of ADHD medication is prevalent, particularly amongst ADHD youth, with only 39% (36–42) remaining on treatment one year after initiation [32].

The period from 15 to 29 years represents a pivotal developmental transition for ADHD youth. While existing systematic reviews of qualitative studies have explored specific dimensions of their lived experience such as peer relationships and friendships [33] bullying and depression [34], resilience factors [15], and teacher-student dynamics [35] these studies focus narrowly on isolated specific dimensions. Yet, the transition to adulthood presents a complex interplay of academic, social, occupational and psychological challenges that cannot be fully understood through fragmented perspectives. By synthesising qualitative studies that span multiple life domains, our review aims to provide a holistic understanding of the unique hurdles faced by ADHD youth during this critical stage. This comprehensive approach will not only illuminate the interconnected nature of their challenges but will also inform the development and evaluation of more effective, tailored interventions and services that address their multifaceted needs as they move into adulthood.

This paper adopts identity-first language for ADHD populations, following guidance from the Irish National Disability Authority [36], the U.S. National Institutes of Health [37], and neurodiverse individuals [38, 39]. Many now reject person-first language (e.g., “youth with ADHD”) as it implies a need to separate from one’s disability. Identity-first language highlights that disabilities arise from societal barriers, advocating for inclusivity. Thus, this paper will use the term “ADHD youth”.

## Methods

### Aim

This meta-ethnographic review aims to identify and synthesise primary qualitative studies reporting the experiences and perceptions of ADHD youth aged 15 to 29 years old, across life domains.

### Design

A qualitative evidence synthesis approach, utilising meta-ethnographic methods was employed for its capacity to rigorously synthesise findings from multiple studies, facilitating higher order interpretation and the development of conceptual understandings that extend beyond individual studies, and its suitability to synthesise lived experiences and generate findings that can inform clinical decision-making [40–42]. A study protocol was registered on PROSPERO [CRD42024544252]. This study is reported in line with the eMERGe meta-ethnography reporting guidelines [43].

### Reflexivity

The lead author (J.T.), a registered occupational therapist and PhD candidate, conducted all stages of the search and analysis under the supervision of K.R. and A.M.M. At the outset, J.T. was a novice qualitative researcher and subsequently completed postgraduate training in qualitative research and meta-ethnography through reputable institutions. The wider research team included psychiatrists (D.A. and M.W.) with clinical experience working with ADHD youth, and academics (K.R. and A.M.M.), both occupational therapists with prior clinical backgrounds and experience in qualitative research and evidence synthesis. J.T. maintained a reflective journal throughout the analysis, which was discussed during supervision to support reflexive awareness of how professional backgrounds and prior experiences might have influenced interpretation.

### Search

A comprehensive search strategy was developed and validated utilising the Peer Review of Electronic Search Strategies (PRESS) [44] (see supplementary file 1), with a university medical librarian, Ms. Elizabeth Dore, acting as the reviewer. A search of ten electronic databases was conducted (APA PsycINFO, APA PsycARTICLES, British Education Index, CINAHL, Embase, ERIC, Medline, PubMed, Web of Science, and Scopus) in April 2024. Three core concepts (ADHD, Youth and Qualitative) and their synonyms were developed into search strings and used to search each database (see supplementary file 2). Existing systematic reviews in this field, and the PRESS informed the creation of synonym sets.

### Inclusion/exclusion criteria

Articles which met the following inclusion criteria were eligible for inclusion:

1. Peer reviewed studies published between 2004 and 2024 that employed recognised qualitative data collection and analysis methods were included. Mixed methods papers were eligible for inclusion where qualitative data could be extracted separately.
2. Studies including participants aged between 15 and 29 years old, or with a mean age within this range, consistent with the European Commission’s definition of youth [12].
3. Studies including participants with either a formal diagnosis of ADHD or self-identified ADHD were eligible, recognising the lengthy waitlists for formal diagnostic assessments worldwide [45] along with the excessive costs often associated with availing of formal supports [46, 47].
4. Studies in which no more than 50% of the participant sample have co-occurring diagnoses. Although co-occurrence of psychiatric disorders is highly prevalent among ADHD populations, this threshold was set to ensure that the findings primarily reflect ADHD-related experiences rather than being disproportionately shaped by other diagnoses.
5. Studies where participants had a dual diagnosis of Autism were excluded, in recognition of the distinct experiences and perspectives associated with Autism, and the significance of Autism/ADHD (AuDHD) diagnoses which warrant independent investigation and focused investigation.

### Screening

Database search results were imported to EndNote X9 software and deduplicated. The remaining papers were exported to Rayyan where the research team screened in two stages, independently and blindly (JT 100%, KR 50%, AM 50%); screening the title and abstract and screening the full text. Disagreements were managed through discussion.

### Sampling

When searching the literature for a meta-ethnographic study, unlike meta-analyses, importance is placed on the sample being purposive and not exhaustive, as the aim is to interpret and explain rather than predict [48]. Consequently, it is not necessary to locate and include every existing study; instead, the value of the synthesis lies in the breadth and depth of concepts and contexts represented across the included studies [49]. Purposive sampling (PS), which is a study selection and sampling method for effectively managing numerous studies that fulfil the inclusion criteria [50] and for preserving the quality of the analysis through refinement of the number of included studies [51, 52] was employed. Following full text review a two-step PS procedure was employed, modelled from the approach described previously by Ames and colleagues [51] (see supplementary file 3);

1. Sampling for maximum variation, in terms of setting and dimension of lived experience studied.
2. Sampling for data richness and for match of study scope.

### Data extraction & analysis

Meta-ethnography is described as a seven-stage process. Phase one involved development of the research question and inclusion criteria and phase two encompassed the search and selection of included studies. In phase three the lead author read and re-read all included articles to extract study characteristics to a custom data extraction table. At this stage relevant findings in included studies were extracted and uploaded to NVivo Version 20.7.2 Pro software. Relevant findings included themes or analysis developed by authors of the included studies and direct quotes from participants in these studies. Study characteristics were extracted from all included papers using a custom data extraction table, with headings: Reference, Title, Year, Country, Recruitment Setting, Research Aim, Sample Characteristics, ADHD Diagnosis, Data Collection, Methods, ADHD Medication, Treatments Engaged In, and Summary of Findings. This process was completed independently by JT, with KR and AM each reviewing 7% of data extraction. In the fourth phase, the key findings from individual studies and their relationship between studies were determined by coding all data uploaded to NVivo inductively. Phase five involved ‘translating the studies into one and other’, utilising constant comparison to highlight similarities and differences between coded concepts from phase 4. Studies were translated into one and other through an iterative, interpretive process of comparing and contrasting. This phase culminated in the development of themes. In phase six a ‘line of argument synthesis’ was created integrating findings across themes. Finally, phase seven involved writing up the synthesis in accordance with the eMERGe checklist (see supplementary file 4) [43].

### Critical appraisal

Methodological quality of included studies was appraised using the Critical Appraisal Skills Programme for Qualitative Studies (CASP). Each paper was independently assessed by two members of the research team (JT 100%, AMM 50%, KR 50%). Disagreements were managed through discussion. No studies were excluded as a result of quality appraisal (see supplementary file 5).

## Results

### Purposive sampling

**Fig. 1:**
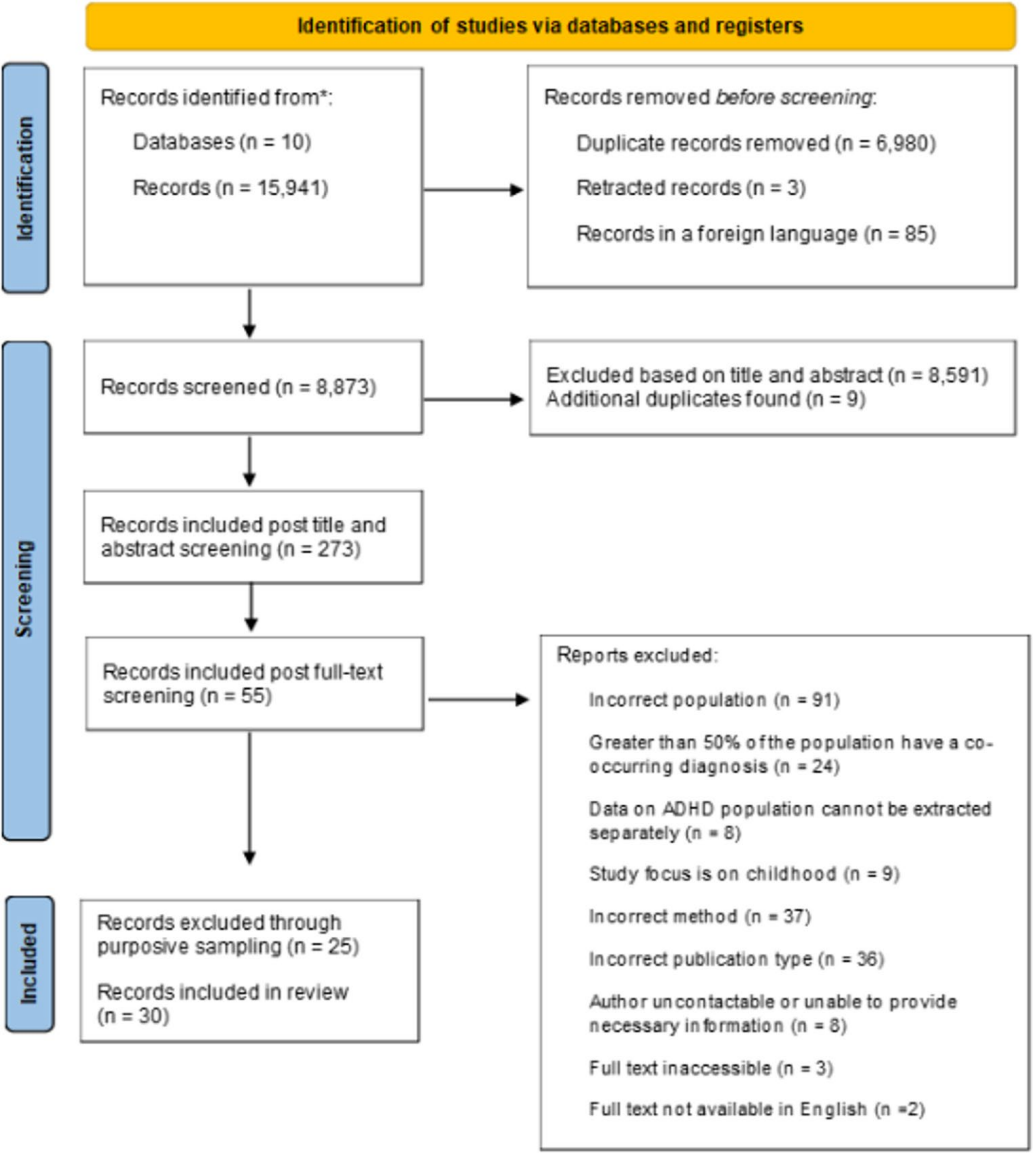
A PRISMA flow diagram illustrates the study selection process [57]

The study selection process is illustrated in Fig. 1. Following full text review fifty-five papers met the study inclusion criteria and these were reviewed to sample for maximum variation by dimension of lived experience. This led to the inclusion of studies which, for example, were the sole study focused on sport [53], weight-management [54] and primary care provision [55]. The studies with the highest (28.6 years) [54] and lowest mean age (15.1 years) [56] were also included as part of this process. The remaining studies were selected due to their data richness and alignment with the study scope. In total thirty papers were selected for inclusion.

### Summary of characteristics

The thirty included studies report the experiences and perspectives of 805 ADHD youths (295 women, 478 men, one non-binary participant, one who preferred not to disclose gender, and 30 participants for whom gender data was not collected/reported). The mean age across studies that reported this information was 21 years. Of the 805 participants across the thirty studies, all but twenty-nine had a formal diagnosis of ADHD. Across studies, medication usage was reported in seventeen studies and revealed three varied patterns of medication use, daily use, occasional use and discontinued use. Non-pharmacological treatments, such as counselling, behavioural therapies, skills training, psychoeducation, and occupational support, were accessed by some or all participants in twenty-one studies (see supplementary file 6).

All included studies were published between 2006 and 2024 and were conducted across eleven countries, including the United States of America (11), Israel (4), the United Kingdom (3), Canada (2), Denmark (2), Norway (2), Korea (1), China (1), Ireland (1), Australia (1), North America (1), and Sweden (1).

Participants were mainly recruited through post-secondary educational settings and healthcare services, alongside liaising with ADHD organisations and utilising online fora. Semi-structured interviews were the most common data collection method, employed by twenty-six studies, one combined semi-structured interviews with focus groups, two used surveys employing phenomenological questions, and one utilised solely focus groups.

The focus areas of included studies can be categorised into experiences and perceptions of education (10), ADHD medication (5), living with ADHD (5), service provision (3), employment (2), emotional dysregulation (1), sport (1), stress (1), weight management (1) and substance use (1).

### Quality appraisal

The results of quality appraisal of included papers are presented in supplementary file 5. No studies were excluded based on their quality, as all contributed valuable and important information regarding the research question, however just 4 of 30 studies received a positive score for every criterion.

### Themes

The analysis generated seven themes, or third-order constructs: (1) Navigating identity and authenticity (2) Diagnosis disclosure dilemma, (3) New ADHD manager reporting for duty, (4) The power of others, (5) Diverse healthcare journeys, (6) Impact on engagement in everyday activities, (7) Turning insight to action: application of self-help strategies. These themes, the number of studies relevant to each theme, and their key concepts can be found in Table 1.

**Table 1:** Themes

Theme	Number of studies contributing to this theme	Key Concepts
Navigating identity and authenticity	13 studies	ADHD as identity; authenticity with/without medication; positive attributes (creativity, energy); struggles with meaning of diagnosis.
The diagnosis disclosure dilemma	11 studies	Stigma; selective disclosure; unmet expectations for support; confidentiality concerns; workplace and sport disclosure dynamics.
New ADHD manager reporting for duty	11 studies	Transition to college; transition to adult services; shifting parental roles; autonomy in treatment decisions; medication discontinuation.
The power of others	22 studies	Supportive peers; non-ADHD relationships; bullying/rejection; impulsivity in interactions; challenges with conflict and social fatigue.
Diverse healthcare journeys	13 studies	Access barriers; long waitlists; differences between child and adult services; medication access issues; patient clinician relationship; perceived provider knowledge gaps.
Impact on engagement in everyday activities	20 studies	Academic challenges; employment barriers; reduced motivation; social and sports participation; sleep and eating difficulties.
Turning insight to action: application of self-help strategies.	13 studies	Strategies to cope with ADHD including movement/fidgeting; physical activity; mindfulness; distraction; avoidance; use of substances or food for regulation.

#### Navigating identity and authenticity

Thirteen papers delve into the impact of ADHD on youths’ identity formation and authenticity. Many youths identify ADHD as integral to their identity and sense of self [58–62], aptly described metaphorically in one study:

*“ADD does not describe me*,* it is me ….it’s not just two or three things about me. It’s like an octopus cause it has tentacles into every single aspect of me …I’m ADD. It affect[s] everything.”* [61].

Some participants reported being unable to even imagine who they would be without ADHD [58–60]. Four studies described youth’s feelings of authenticity when they can experience and express their true identity, unmedicated [56, 58, 60, 63]. In a small number of studies ADHD medication was described as diminishing the exuberance that is part of their identity [56], creating a dilemma: to sacrifice their authenticity by using medication to enhance functioning, or to remain true to their identity going unmedicated [63].

Many studies described the impact of ADHD as a gift while others described it as a flaw negatively affecting their identity [56, 58, 60–62, 64–69]. Some youths appreciated the unique strengths it brings [56], such as creativity [56, 58], energy [58], individuality [66], sensitivity, empathy, insight [61] and outgoing characteristics [61, 62, 66].

Attaining an ADHD diagnosis was described in one study as empowering, providing validation and explanation for day-to-day challenges [64]. However, five other studies reported that youth find little meaning in attaining a diagnosis due to doubt surrounding its validity [56, 59, 62, 68, 69]. Furthermore, some youths struggle to connect their everyday experiences with ADHD characteristics [56, 59, 68], as described by one youth who does not experience inattention when the topic interests them:

*“an ability not to have an attention span for very long. But I can have an attention span for extremely long for the things that I care about.”* [68].

#### The diagnosis disclosure dilemma

Eleven papers detail the complexities of disclosing ADHD [53, 56, 59, 61, 62, 64, 66, 70–74]. Youth report refraining from disclosing due to negative associations and stigma [62, 64, 66, 71, 72] gaining a sense of control by selectively sharing their diagnosis [70] or seeking to protect themselves from misunderstanding, judgement and rejection [64]. This creates a dilemma:

*“There is a dilemma between keeping ADHD a secret to prevent stigmatisation*,* and at the same time longing for understanding from peers*,* colleagues and superiors.”* [72].

Some youths chose not to disclose in education settings as they did not feel they required assistance [71] or because they wanted to be treated the same as classmates [74]. While youth may disclose their diagnosis with the expectation of receiving supports or accommodations [71, 74] this is not always realised. Two studies describe challenges obtaining formal supports in university settings [61, 71]. In two studies, youth grappled with disclosure due to the risk of their confidentiality being compromised to classmates [61, 65].

*“One time I even got [my accommodation letter] out before class and three different people passed it around*,* and I was like*,* “Excuse me. Hello. That’s mine”. Yeah*,* they’re [sic] like*,* “Ooh*,* what’s this? Ooh*,* I need to get me one of these forms. Then I’ll be a good student.””* [61].

Two studies described disclosure in employment settings leading to youths’ ADHD characteristics being viewed positively [59] and receiving understanding and support from colleagues and superiors [72].

In team sports, youth felt compelled to disclose their diagnosis to coaches and teammates, to prevent misunderstandings and foster compassion [53]. Describing disclosure to coaches and teammates one participant reported:

*“I am very verbal about it*,* having the problem. I let people know. Definitely. Because if you don’t let them know they are just going to be on you.”* [53].

Diagnosis disclosure in friendships varied from those who openly share their ADHD diagnosis with friends [56, 61, 73], to those who feel comfortable only confiding in close friends [62] and in one study some participants reported they prefer to keep their diagnosis private [61].

#### New ADHD manager reporting for duty

Eleven articles document youth’s experiences and perceptions of transitions from adolescent to adult [56, 60–62, 65, 69, 74–78] inclusive of experiences of transition from school to college [60–62, 69, 74, 76–78] and from child to adult health services [56, 60, 75, 77].

Entering college can be unsettling for youths [69], as they face a gradual increase in academic demands and responsibility for managing ADHD independently without parental support, which some perceived as a reasonable reflection of their maturation and growing independence [62, 74], while others enter college feeling unprepared to manage ADHD independently [62]. Parents who advocated for the youth by disclosing disabilities to teachers and assisting with schoolwork, offered immediate support but hindered their development of self-advocacy skills [62] with some youth in college just beginning to understand the importance of self-advocacy skills and their role in managing ADHD [74] and some feeling shocked when responsibilities shift [77].

*“And now I’m like trying to take care of this all on my own just for me like just to take care of my own self I’m like wow*,* like it’s I didn’t realize like how much it is to deal with life with ADHD.”* [77].

Similarly, the transition from child to adult healthcare services presents increasing responsibilities and challenges, with youth having varied perceptions of their readiness [60]. Their sense of preparedness is shaped by their knowledge of the process, feelings about adult services, and familiarity with them [60]. This shift grants increased autonomy over their ADHD treatment [56, 65], moving from passive participants to active decision makers [65], with two studies describing how this may result in unassisted discontinuation of ADHD medication [56, 75].

*“When they became adults*,* neither psychiatrists nor parents could control their decisions related to medication*,* and many of the participants chose to discontinue medication on their own*,* without involving anyone else.”* [75].

#### The power of others

Twenty-two articles report youths’ experiences and perceptions of navigating social relationships with ADHD [53, 54, 56, 58, 59, 61–64, 66, 67, 70, 72–81].

For those who formed supportive social connections with ADHD peers, the reported benefits were significant [59, 64, 66, 67, 73, 75, 77, 78] such as feelings of support and validation. In college, the openness of ADHD peers regarding their experiences deepened their understanding of ADHD [73]. One notable benefit was the opportunity to offer advice to fellow ADHD peers, as illustrated by this reflection:

*“(…) I want to help others to feel as good as I do now (…). I have a friend*,* who sent me a message asking about my experience*,* and if I had any advice that I could give her. Then*,* I felt I was useful to her.”* [67].

Social connection with non-ADHD friends, romantic partners, roommates, teammates, coworkers and classmates were also positively described, helping to mitigate the impact of ADHD on daily life [53, 54, 63, 64, 72–74, 78, 80]. These relationships were described as providing essential support, for example, college student peers provided reminders about deadlines and supported study [73, 78] and relationships with coworkers could boost motivation and enable task initiation [72, 78].

However, despite these reported benefits, some reported feelings of isolation [59, 64, 72, 77], misunderstanding from non-ADHD peers [64] and exclusion through rejection, bullying or microaggressions which they perceive as a consequence of their ADHD [53, 59, 64–66]. Bullying was reported across four studies including physical or emotional bullying from peers, classmates and from medical professionals [59, 64–66].

Many youths perceive ADHD to negatively affect their social skills and relationships, leading to inappropriately “blurting out comments”, being “blunt”, saying “something impulsive”, or “tactlessness” [53, 61, 66, 73, 74, 80]. Three studies detailed experiences of interrupting conversation [53, 61, 80] due to boredom, fragmented thinking [61] or to release internal tension [80], leading to immediate regret for some [53, 80].

*“I will say something impulsive and then realise*,* “oh god*,* Megan why did you do that?” and feel bad. When I’m impulsive I always feel regret. It’s not thrilling*,* it’s just stupid. I lose it and then feel regret.”* [80].

Three studies report challenges managing conflict in relationships [76, 80, 81] and process of establishing and tending to relationships was also commonly described as overwhelming, leading some to worry about and/or withdraw from social connections [64, 76, 81].

#### Diverse healthcare journeys

Thirteen studies report on ADHD youths’ healthcare journeys. Perceived ease or difficulty in accessing or engaging with ADHD-related services was reported in studies [55, 59, 60, 62, 65, 67, 70, 75, 82]. Positive experiences included the availability of numerous services for managing ADHD and co-occurring conditions [82], easy access to services, continuity of care from childhood to adulthood [65] and timely service cessation [60].

However, barriers to accessing services were also commonly reported [55, 59, 60, 62, 70, 82]. Some had to access private services because schools failed to refer them for diagnosis [82] or due to long waiting lists for public services [55]. Young women with ADHD in two studies noted that they are skilled in masking symptoms, and healthcare professionals may overlook symptoms in girls/women [55, 59].

Other challenging experiences included limited access to treatments suited to their needs [75] and narrow service remits failing to consider all aspects of health [60].

Accessing pharmacological treatment was described as arduous in three studies [55, 60, 70] for example difficulty attaining repeat prescriptions or changing medication dosage [55].

Seven studies discussed youths’ relationships with healthcare professionals [55, 59, 60, 65, 72, 82]. While some youths described positive relationships, others reported a lack of connection [65] or felt unheard in clinical encounters [60]. One study reported a distinction between the caring approach of clinicians in child services compared to those in adult services;

*“A little bit more like ‘How are you going to cope with your exams? Have you spoken to your school to get extra time?’ But I feel like in the adult one it’s a little bit less*,* it’s like you need to just find out for yourself kind of thing.”* [60].

Several studies described varied levels of information on ADHD and treatment options provided by health professionals [55, 60, 65, 71, 73, 75, 82]. In some cases, youth felt providers lacked knowledge and needed neurodiversity and ADHD training [55, 61, 65]. Due to a lack of information from providers many youths conducted their own research or gained information from ADHD peers [73, 75].

*“So I do feel I just need to be generally more informed about ADHD*,* about the services that are on offer and that would be much more useful to me*,* because now. well I do accept*,* with some resentment*,* that this is something that I will have to deal with for the rest of my life*,* so if that’s the case then I want to be as prepared as I can be.”* [60].

#### Impact on engagement in everyday activities

Twenty studies report on the impact of ADHD on youths’ engagement in everyday activities, namely education, employment and leisure. Nine studies report ADHD youth doubt their ability to succeed in education, perform sub-optimally, experience struggles with motivation, concentration and deadlines, and perceive education as ableist [61–63, 66–68, 74, 77, 78].

*“I can’t do what I want so*,* I just never take that extra step to try. I’m like what’s the point of putting all this hard work into it if I’m not*,* if I don’t see or I don’t feel*,* feel the good feelings when you succeed.”* [78].

Youth report encountering barriers to employment as a result of ADHD, including challenges in job searching, securing suitable positions, and completing work-related tasks [59, 62, 64, 65, 68, 69, 72, 78] exemplified by a youth’s experience with their employer:

*“ “You’re very fun to work with” But she just says*,* “This*,* you know*,* this place just doesn’t seem to be for you. Your strengths can be better applied elsewhere.””* [68].

Studies report youth searching for employment often face low self-esteem and confidence, leading to fears of rejection, and reduced motivation, even for desirable positions [72, 78]. A high-interest in the field and engaging work tasks was characterised as good-fit employment for ADHD youth [64, 72, 78]. Employed youth commonly reported dissatisfaction due to workplace boredom, particularly in uninteresting tasks, which they attribute to their ADHD-related challenges [64, 65, 78].

ADHD impacts various leisure activities, including team sports [53, 56, 73, 78], social activities [63] and willingness to try new leisure activities due to anticipating failure of fearing embarrassment [78]. During sports ADHD can lead youth to become sidetracked or distracted [53].

*“It’s not about knowing what to do. It’s about not doing what one knows because of focus issues. I have all these skills in the world but I can’t really put it into use because at times I am doing things I shouldn’t be doing on the field because of my lack of focus.”* [53].

Across some studies ADHD was reported to have adverse effects on daily eating habits [54, 63, 76] including inattentiveness to hunger signals [63], irregular eating patterns, binge eating [76] emotional eating and overeating [54].

Five studies highlight the impact of ADHD on sleep, with youth reporting difficulties falling asleep, frequent daytime napping, and challenges waking up in the morning [63, 66, 73, 76, 78, 80]. Their sleep often feels unrefreshing, leaving them in a constant state of exhaustion, affecting other activities and fostering feelings of failure that they are wasting their time [63, 76].

*“Sleep*,* which should clear my head*,* does not help me relax and does not charge my battery. It takes me a lot of time to fall asleep and then it’s difficult for me to wake up…. I sleep very little and most of time I am very tired*,* which affects my performance in class…”* [63].

#### From insight to action: application of self-help strategies

Thirteen papers describe the use of self-help strategies to manage ADHD symptoms or associated challenges [53, 54, 62, 64, 66, 67, 69, 73, 75, 78–80, 83].

Across five studies youth describe movement, such as fidgeting, and physical activity as useful to independently manage their ADHD symptoms, address emotional dysregulation [53, 66, 78, 80, 83] provide an outlet for nervous energies, and improve focus [53], as explained by one youth.

*“I’m kicking my leg instead of starting to cry or instead of getting overwhelmed by what I’m talking about*,* like it just calms me.”* [80].

Mindfulness and relaxation approaches, such as yoga and breathwork were utilised by youths in four studies with benefits for re-directing and controlling their attention, behaviour and emotions reported [53, 64, 66, 78].

*“Well*,* a little bit ago when I was with a girl last year*,* we went into this whole meditation and yoga bit and that was really helpful too… Like to just clear your head a little bit and focus on your breathing*,* and that helps a lot.”* [78].

Some youth practiced avoidance and distraction from the immediate situation through schoolwork, listening to music or watching video clips, to calm themselves down [62, 80, 83].

Youths in five studies reported the use of alcohol, smoking, other substances and food as a self-help strategy [54, 64, 69, 73, 79, 83] to enhance concentration, provide calming effects [73], and address emotional dysregulation [83].

## Line of argument synthesis

A line of argument was developed to integrate the seven themes and offer a fuller picture of ADHD youths’ lived experiences and perceptions. Across the findings, a unifying insight emerged: the challenges faced by ADHD youth often stem more from societal structures, norms, and expectations than from ADHD itself. Some ADHD youth explicitly articulated a belief that their ADHD stems from societal and environmental factors. Others implied that society contributes to negative experiences and barriers, such as the pressure to appear “normal”. Many felt their authentic ADHD selves were unaccepted by society, leading to recommendation of pharmacological intervention aimed at conformity. Participants also reported feelings of overwhelm from trying to meet societal expectations, alongside experiences of social exclusion, including rejection, bullying, and microaggressions related to their ADHD. Collectively, these perspectives suggest that the difficulties encountered by participants are often socially constructed, reflecting a mismatch between individual neurodiversity and societal expectations rather than deficits inherent to ADHD.

This line-of-argument synthesis aligns with the International Classification of Functioning, Disability and Health (ICF), which conceptualises functioning as the dynamic interaction between an individual’s health condition and contextual factors, including environmental and social influences [84]. The finding that many challenges faced by ADHD youth arise not from ADHD itself but from societal norms, expectations, and barriers reflects the ICF’s emphasis on environmental factors as determinants of participation.

## Discussion

Through meta-ethnographic analysis of findings, this study generated a rich, contextualised understanding of the experience of ADHD youth across life domains. Thirty studies reporting the experience of 805 ADHD youth across eleven countries were included. Findings revealed the complexity of this developmental stage. While some individuals view ADHD as core to their identity and conferring benefits, many face significant challenges impacting their transition to independence - particularly in managing social relationships. Participants reported a delicate balance of disclosing their diagnosis with the need for support and understanding in education, employment, and social settings.

A key finding of this qualitative evidence synthesis was that ADHD youth emphasise its centrality to their identity, highlighting positive attributes including creativity, energy, individuality, sensitivity, empathy and insight. This aligns with a substantial body of qualitative and quantitative research identifying strengths associated with ADHD [5, 6, 67]. Such perspectives are also reflected in online ADHD communities, where it is common for individuals to assert agency in defining what ADHD means to them, and share positive narratives that contribute to the de-medicalisation of ADHD [85]. However, this strengths-focused perspective is not universal. Some parents of ADHD children express annoyance at ADHD being positively portrayed, perceiving it to minimise their struggles of parenting an ADHD child [86], alongside some ADHD children self-reporting strengths within the typical range and consistent with non-ADHD peers [87] not viewing themselves as encompassing additional strengths due to ADHD. Clinically, an emphasis on strengths is increasingly encouraged. Strengths-based approaches following diagnosis and during treatment planning are endorsed by the Australian evidence-based clinical practice guideline for ADHD [88]. Parents of autistic adolescents’ report that strengths-based interventions can foster belonging and confidence in their adolescent whilst positively impacting health, wellbeing and social relationships [89]. Furthermore, ADHD adults prefer, and even opt for private, strengths-based interventions over traditional deficit-focused public mental health care [90]. This indicates increasing recognition that ADHD encompasses both challenges and positive attributes, and that strengths-based approaches may offer clinical value for adolescents, youth, and adults.

We found that the difficulties encountered by participants are often socially constructed, reflecting a mismatch between individual neurodiversity and societal expectations rather than deficits inherent to ADHD. This aligns with the Social Model of Disability (SMoD) [91] which suggests that the challenges faced by disabled people arise from social oppression and exclusion, rather than personal deficits [91–93], emphasising the need to remove of social, attitudinal and environmental barriers to participation [93, 94]. Findings revealed that the experiences of ADHD youth are strongly influenced by societal factors, particularly social relationships and the expectations and responses of peers. Although some youth did report supportive peers who provide validation and help mitigate the impact of ADHD in daily life, many others experienced social isolation, bullying, and misunderstanding from non-ADHD peers, along with social skill challenges that hindered their relationships. A review of ADHD children’s social behaviour argues that it is not that this population lack interest in social connections, rather they experience difficulty adjusting to the behaviours of others [95], or conforming. Studies indicate that ADHD youth have fewer friends, lower quality friendships and poorer friendship interventions compared to typically developing peers and further research assessing the nature of friendships over time is recommended [96]. Clinicians have been advised that assessment of ADHD individuals should examine social functioning, with a particular focus on the quality of friendships [97], and although several clinical peer functioning interventions exist, they should be modified, to have a long-term intensive ADHD focus on peer functioning at home and school, and to involve parents to increase positive outcomes [33].

Consistent with our findings, numerous studies have reported that ADHD youth, their parents and healthcare professionals describe the transition from child to adult health services as fragmented and poorly co-ordinated, with parents often feeling excluded from clinician communication and sidelined compared to their prior involvement [98–100]. In response, protocols and recommendations have been established to facilitate a successful transition to adult services for all involved with recommendations such as appointing a clinician who is familiar to the youth as the transition lead [3, 101, 102]. Notably this qualitative evidence synthesis found that some youth report a lack of connection with their clinicians, discontinuity of care, and exclusion from treatment decisions. Existing literature highlights the importance of the patient-clinician relationship (PCR) in influencing how individuals experience the transition to adult mental health care [100]. The PCR directly influences shared decision-making (SDM), whereby the clinician and patient make decisions together, essential for fostering patient autonomy [103]. Across diagnoses, healthcare users report barriers to SDM stemming from poor PCRs - marked by care discontinuity, limited personal connection, authoritarian clinician behaviour [104] and systemic issues such as long waiting times and brief appointments [105, 106]. This echoes the experiences of ADHD youths within this qualitative evidence synthesis. Additionally, for youth with mental health difficulties there may be unique challenges to SDM involving clinicians, youth [107] and parents [108]. While various approaches are being developed to support SDM in child and youth mental health such as the use of decision aids, rigorous research evaluating the effectiveness of these approaches is largely lacking [109] and research on parent preferences for decision support interventions is also scant [108]. Given this, interventions that jointly target both clinicians and patients represent a particularly promising avenue for embedding SDM into routine clinical practice [110].

This study found that ADHD youth frequently face additional challenges during the transition to third-level education. They experience self-doubt regarding their educational success, face motivation and concentration barriers, and find the education system to be unaccommodating. These struggles are intensified as the youth are required to self-manage their ADHD and take ownership of their treatment, all whilst entering new life contexts. A quantitative exploration of medication adherence during the transition to college suggested that this is a high-risk period for poor self-management of ADHD, with participants adhering to just 53.53% of prescribed medication dosages [111]. Furthermore, an exploration of medication adherence in ADHD children and adolescents found an association between non-adherence and increasing age, due to desires for autonomy, stigma and negative attitudes toward medication [112]. In the transition to the employment domain, ADHD youth within this qualitative evidence synthesis report barriers to and within employment, fear of rejection when applying for roles, low self-esteem, and dissatisfaction and boredom in employment as a result of their ADHD. Youth with a history of ADHD hold lower ranking and paying employment positions with increased financial dependence on the family [22]. A consensus statement from health professionals found that ADHD adults require support during all stages of employment, from completing and submitting applications, to interviewing and disclosure decision-making [113]. This qualitative evidence synthesis and available literature collectively reveal that ADHD youth and adults encounter significant challenges when transitioning to university and employment and highlights the critical need for effective supports and interventions to facilitate effective self-management and promote successful transitions for ADHD youth.

### Strengths and limitations

A rigorous qualitative approach, broad search strategy, and ongoing reflective engagement with the studies ensured the validity, rigor, and transparency of our findings. To the best of the research team’s knowledge, this meta-ethnography is the first to synthesise the lived experiences and perceptions of ADHD youth aged 15 to 29 years old, worldwide across life domains.

Limitations of this study may affect the generalisability of findings. The search strategy was restricted to studies published in the English language. Although the search strategy was comprehensive it is possible that relevant studies were missed due to human error, or the difficulties associated with searching for qualitative studies. We excluded studies where more than 50% of participants had a co-occurring diagnosis or where there was a dual Autism/ADHD diagnosis to maintain a primary focus on ADHD-related experiences. However, this approach may have constrained the breadth of our findings by excluding studies that could have offered valuable insights into how ADHD intersects with other psychiatric or neurodevelopmental conditions. As co-occurrence is highly prevalent among individuals with ADHD, limiting the representation of these experiences may reduce the extent to which our synthesis captures the full complexity of living with ADHD in the context of additional diagnoses. Furthermore, studies focusing on perceptions of interventions and treatments were excluded, as they did not align with the aim of our study. However, they provide valuable data which should synthesised in the future. No study was excluded based on quality appraisal, however just 4 of 30 studies received a positive score for each CASP criterion and the majority did not adequately consider the relationship between the researcher and participant. Qualitative research findings are regarded as a shared product of the researcher and participant relationship, which when examined affords the researcher the opportunity to demonstrate reflexivity, enhancing the integrity and trustworthiness of the findings [114]. Future qualitative studies may benefit from implementation of formal reporting guidelines, such as the Consolidated Criteria for Reporting Qualitative Research, which emphasises and facilitates researcher reflexivity and the reporting of such processes [115]. The included studies employed a wide range of qualitative methodologies and analytic approaches, spanning descriptive, interpretative, and constructivist traditions. Such methodological diversity may limit comparability across studies and could influence the coherence and robustness of the overarching conclusions developed in this synthesis.

## Conclusion

This qualitative evidence synthesis aimed to comprehensively describe the lived experiences and perceptions of ADHD youth. Across 30 studies, youths reported how ADHD was central to their identity, led to dilemmas about disclosing their diagnosis, and had significant implications for their relationships and the challenges they faced in education, work, daily life, and accessing support. Many difficulties were attributed less to ADHD itself and more to societal expectations and structural barriers. Youths also described a range of self-directed coping strategies, highlighting both the complexity of this transitional life stage and the adaptive ways they navigate it. Future research, building on the findings of this synthesis, should prioritise the development and evaluation of interventions tailored to the unique transitional challenges faced by ADHD youth, particularly during key shifts into higher education, employment, and adult healthcare. Interventions that strengthen self-management, support disclosure decision-making, enhance social functioning, and embed shared decision-making within clinical care are especially needed.

## Supplementary Information

Below is the link to the electronic supplementary material.

Supplementary File 1 (PDF 144 KB)

Supplementary File 2 (PDF 177 KB )

Supplementary File 3 (PDF 350 KB)

Supplementary File 4 (PDF 165 KB)

Supplementary File 5 (PDF 242 KB)

Supplementary File 6 (PDF 242 KB)

## Data Availability

All datasets used and/or analysed within this study are freely available from the corresponding author upon request.
