# Supplementary material for: “It has tentacles into every single aspect of me” a qualitative evidence synthesis of the lived experiences and perceptions of ADHD youth
Source: Eur Child Adolesc Psychiatry. 2026 Feb 25;35(5):1435–49. doi: 10.1007/s00787-025-02955-8 (PMC13272611; doi:10.1007/s00787-025-02955-8)
Supplement: Supplementary file 1 — (PDF 144 KB) [file 787_2025_2955_MOESM1_ESM.pdf]

“It has tentacles into every single aspect of me” A Qualitative Evidence Synthesis of the Lived Experiences and Perceptions of ADHD Youth.

European Child & Adolescent Psychiatry

Jessie Tierney[1], Health Research Institute, School of Allied Health, Faculty of Education and Health Sciences, University of Limerick, Ireland. tierney.jessie@ul.ie

Doctor Ann-Marie Morrissey[2], Ageing Research Centre, Health Research Institute, School of Allied Health, Faculty of Education and Health Sciences, University of Limerick, Ireland.

Doctor Dimitrios Adamis[3], Sligo Mental Health Services Adult ADHD Clinic, Sligo, Ireland; and Department of Psychiatry, University of Limerick, Ireland.

Doctor Margo Wrigley[4], HSE National Clinical Programme for ADHD in Adults, Health Service Executive, Dublin 8, Ireland.

Doctor Katie Robinson[2], Ageing Research Centre, Health Research Institute, School of Allied Health, Faculty of Education and Health Sciences, University of Limerick, Ireland.

Peer Review of Electronic Search Strategies Framework

Appendix A

***PRESS Guideline* — Search Submission & Peer Review Assessment**

SEARCH SUBMISSION: THIS SECTION TO BE FILLED IN BY THE SEARCHER

Searcher: Jessie Tierney	Email: Tierney.Jessie@ul.ie	
Date submitted:	Date requested by:	*[Maximum = 5 working days]*

Systematic Review Title:

A Qualitative Synthesis of Primary Studies Concerning Youth Experiences and Perceptions of Living with ADHD (Attention Deficit Hyperactivity Disorder).

This search strategy is …

X	My PRIMARY (core) database strategy — First time submitting a strategy for search question and database
	My PRIMARY (core) strategy — Follow-up review NOT the first time submitting a strategy for search question and database. If this is a response to peer review, itemize the changes made to the review suggestions
	SECONDARY search strategy— First time submitting a strategy for search question and database
	SECONDARY search strategy — NOT the first time submitting a strategy for search question and database. If

	this is a response to peer review, itemize the changes made to the review suggestions

Database (i.e., MEDLINE, CINAHL…): *[mandatory]*

Electronic databases that will be used are as follows: APA PsycINFO, APA PsycARTICLES, CINAHL, British Education Index, EMBASE, ERIC, MEDLINE, PubMed, Scopus, Web of Science.

Interface (i.e., Ovid, EBSCO…): *[mandatory]*

EBSCO

Research Question (Describe the purpose of the search) *[mandatory]*

1. Synthesising the lived experiences and perceptions of youth with ADHD.

(a) Inclusive of, but not limited to, their experiences and perceptions of socialisation, education, self-care, employment, leisure, mental health and wellbeing.

PICO Format

(Outline the PICOs for your question — i.e., Patient, Intervention, Comparison, Outcome, and Study Design — as applicable)

For this qualitative evidence synthesis of primary studies, the author adopted a qualitative variation of PICO; Population/Problem, Interest, COntext.

**P**	Youth aged 15-29 years old, with formally diagnosed or self-reported ADHD (See Appendix A).
**I**	Experience(s) and perception(s) of living with ADHD, inclusive of, but not limited to, their experiences and perceptions of socialisation, education, self-care, employment, leisure, mental health and wellbeing.
**CO**	Exploration of experiences and perceptions worldwide, within the last two decades, through synthesis of primary studies detailing youth experience(s) and perception(s) of living with formally diagnosed, or self-reported ADHD.

Inclusion Criteria (List criteria such as age groups, study designs, etc., to be included) *[optional]*

Where majority of the sample (>50%) have comorbidities, the paper will be excluded. Rationale is that whilst many ADHD youth will encounter comorbid diagnoses i.e. depression/anxiety, and we wish to include them in the sample and show recognition of such, if there were to be a majority with an additional diagnosis it may skew results.

Where there is a mixed sample, for example n = 12 ADHD, n = 6 ASD., it will be included if the responses of those with ADHD can be extracted separately.

Inclusion

Published between 2004-2024
In English.
Population aged 15-29 years old, or where there is a mean age of between 15-29 years.
Papers from all countries of origin.
Youth with formally diagnosed ADHD or self-identified ADHD symptoms.
Papers that are qualitative primary studies, using recognised methods of qualitative data collection and analysis
Mixed methods papers, where the qualitative data can be extracted separately.
Peer-reviewed papers.

Exclusion Criteria (List criteria such as study designs, date limits, etc., to be excluded) *[optional]*

Was a search filter applied?

No

If YES, which one(s) (e.g., Cochrane RCT filter, PubMed Clinical Queries filter)? Provide the source if this is a published filter. ***[mandatory if YES to previous question — textbox]***

N/A

Other notes or comments you feel would be useful for the peer reviewer? ***[optional]***

Existing systematic reviews in this field informed creation of the search string and key search terms for this study.

Results of database searches will be imported to EndNote, where deduplication will occur, then exported to Rayyan where the research team will screen results by title and abstract. At this stage all search results will be reviewed by two members of the research team and their eligibility assessed against the above inclusion criteria. Reviewer one (Jessie Tierney) will review 100% of papers within this meta-ethnography. Reviewer two (Katie Robinson) and reviewer three (Ann-Marie Morrissey) will each review 50% of papers. This process will be completed blind. Where there is a disagreement between reviewers surrounding inclusion and/or exclusion of a paper, a verbal discussion will be carried out with all members of the team present to reach consensus.

The full text of all potentially relevant studies following title and abstract screen will be retrieved and read in full and their eligibility assessed against the above inclusion criteria assessed by two members of the team. Reviewer one (Jessie Tierney) will review 100% of papers. Reviewer two (Katie Robinson) and reviewer three (Ann-Marie Morrissey) will each review 50% of papers. This process will be completed blind. Where there is a disagreement

between reviewers surrounding inclusion and/or exclusion of a paper, a verbal discussion will be carried out with all members of the team present to reach consensus. A record of every stage of this process will be maintained.

Papers will be imported to EndNote, where deduplication will occur, then exported to Rayyan where the research team will review and vote for paper inclusion/exclusion.

Please copy and paste your search strategy here, exactly as run, including the number of hits per line.

Search terms and their variations:

Core Search Term	*Synonyms*
ADHD (TI)	ADHD OR ‘attention deficit disorder’ OR ‘attention deficit disorder with hyperactivity’ OR ‘attention deficit hyperactivity disorder’ OR ‘attention deficit’ OR ‘attention-deficit/hyperactivity’ OR ‘attention-deficit hyperactivity disorder’ OR ‘attention deficit and disruptive behavio*’ OR attention* OR ADD OR ‘neurodevelopmental disorder’
Youth (TI OR AB)	youth* OR ‘young person’ OR ‘young adult*’ OR ‘young people’ OR minor OR teen* OR student* OR undergrad* OR adolescen*
Qualitative (TI OR AB)	qualitative OR ethnograph* OR narrative OR interview OR experience* OR ‘thematic analysis’ OR ‘content analysis’ OR ‘mixed method’ OR phenomenolog* OR ‘focus group’ OR ‘case studies’ OR ‘evaluation methods’ OR ‘naturalistic observation’ OR ‘participant observation’ OR ‘social science research’ OR transcript* OR grounded OR ‘purposive sample’ OR ‘discourse analysis’

Appendix A.

Population Inclusion and Exclusion Criteria

Youth aged 15-29 years old [1] with clinically diagnosed ADHD/self-reported ADHD symptoms.

<u>Youth</u>: Eurostat, a European Union hub for high-quality and up-to-date statistics regarding populations within Europe provides a lengthy rationale alongside numerical and categorical definitions for ‘youth’. ‘Youth in Europe’, a book that creates a statistical portrait of this population through use of social, educational and cultural statistics, ‘youth’ is considered 15 to 29 years old [1].

<u>Self-reported ADHD:</u> An individual who self-identifies as having ADHD or self-declares experiencing ADHD symptoms, inclusive of completing self-report scales.

Inclusion:
● Individuals with formally diagnosed ADHD

- Individuals with self-reported ADHD symptoms
- Aged between 15-29 years old
- Mean age between 15-29 years old where mixed age groups are studied
- Members of all ethnicities/countries

1. Eurostat (2009). *Youth in Europe: A statistical portrait*. Office for Official Publications of the European Communities. https://ec.europa.eu/eurostat/documents/3217494/5716032/KS-78-09-920-EN.PDF/1e9dd987-1471-4a05-b449-46c81bc1766c
