# Supplementary material for: “It has tentacles into every single aspect of me” a qualitative evidence synthesis of the lived experiences and perceptions of ADHD youth
Source: Eur Child Adolesc Psychiatry. 2026 Feb 25;35(5):1435–49. doi: 10.1007/s00787-025-02955-8 (PMC13272611; doi:10.1007/s00787-025-02955-8)
Supplement: Supplementary file 2 — (PDF 177 KB ) [file 787_2025_2955_MOESM2_ESM.pdf]

“It has tentacles into every single aspect of me” A Qualitative Evidence Synthesis of the Lived Experiences and Perceptions of ADHD Youth.

Jessie Tierney[1], Health Research Institute, School of Allied Health, Faculty of Education and Health Sciences, University of Limerick, Ireland. tierney.jessie@ul.ie

Doctor Ann-Marie Morrissey[2], Ageing Research Centre, Health Research Institute, School of Allied Health, Faculty of Education and Health Sciences, University of Limerick, Ireland.

Doctor Dimitrios Adamis[3], Sligo Mental Health Services Adult ADHD Clinic, Sligo, Ireland; Department of Psychiatry, University of Galway, Ireland; and Department of Psychiatry, University of Limerick, Ireland.

Doctor Margo Wrigley[4], HSE National Clinical Programme for ADHD in Adults, Health Service Executive, Dublin 8, Ireland.

Doctor Katie Robinson[2], Ageing Research Centre, Health Research Institute, School of Allied Health, Faculty of Education and Health Sciences, University of Limerick, Ireland.

Search Strategy (CINAHL Example)

S1	TI	ADHD OR ‘attention deficit disorder’ OR ‘attention deficit disorder with hyperactivity’ OR ‘attention deficit hyperactivity disorder’ OR ‘attention deficit’ OR ‘attention-deficit/hyperactivity’ OR ‘attention-deficit hyperactivity disorder’ OR ‘attention deficit and disruptive behavio*’ OR attention* OR ADD OR ‘neurodevelopmental disorder’
S2	TI OR AB	youth* OR ‘young person’ OR ‘young adult*’ OR ‘young people’ OR minor OR teen* OR student* OR undergrad* OR adolescen*
S3	TI OR AB	qualitative OR ethnograph* OR narrative OR interview OR experience* OR ‘thematic analysis’ OR ‘content analysis’ OR ‘mixed method’ OR phenomenolog* OR ‘focus group’ OR ‘case studies’ OR ‘evaluation methods’ OR ‘naturalistic observation’ OR ‘participant observation’ OR ‘social science research’ OR transcript* OR grounded OR ‘purposive sample’ OR ‘discourse analysis’
