# Supplementary material for: “It has tentacles into every single aspect of me” a qualitative evidence synthesis of the lived experiences and perceptions of ADHD youth
Source: Eur Child Adolesc Psychiatry. 2026 Feb 25;35(5):1435–49. doi: 10.1007/s00787-025-02955-8 (PMC13272611; doi:10.1007/s00787-025-02955-8)
Supplement: Supplementary file 3 — (PDF 350 KB) [file 787_2025_2955_MOESM3_ESM.pdf]

“It has tentacles into every single aspect of me” A Qualitative Evidence Synthesis of the Lived Experiences and Perceptions of ADHD Youth.

European Child & Adolescent Psychiatry

Jessie Tierney[1], Health Research Institute, School of Allied Health, Faculty of Education and Health Sciences, University of Limerick, Ireland. tierney.jessie@ul.ie

Doctor Ann-Marie Morrissey[2], Ageing Research Centre, Health Research Institute, School of Allied Health, Faculty of Education and Health Sciences, University of Limerick, Ireland.

Doctor Dimitrios Adamis[3], Sligo Mental Health Services Adult ADHD Clinic, Sligo, Ireland; and Department of Psychiatry, University of Limerick, Ireland.

Doctor Margo Wrigley[4], HSE National Clinical Programme for ADHD in Adults, Health Service Executive, Dublin 8, Ireland.

Doctor Katie Robinson[2], Ageing Research Centre, Health Research Institute, School of Allied Health, Faculty of Education and Health Sciences, University of Limerick, Ireland.

Purposive Sampling Tables

Papers for Inclusion

Author	Year	Score	Comment
Ben-Dor Cohen	2023	3	A reasonable amount of qualitative data that relate to the synthesis objective.
Brinkman	2012	4	A good amount and depth of qualitative data that relate to the synthesis objective.<br>Also, maximum variation – Population (age).
Chen	2023	5	A large amount and depth of qualitative data that relate in depth to the synthesis objective.
Cheung	2015	N/A	Maximum variation – Country.
Clancy	2020	5	A large amount and depth of qualitative data that relate in depth to the synthesis objective.
Darby	2023	5	A large amount and depth of qualitative data that relate in depth to the synthesis objective.
Druedahl	2018	5	A large amount and depth of qualitative data that relate in depth to the synthesis objective.
Fleishmann	2017	5	A large amount and depth of qualitative data that relate in depth to the synthesis objective.
Godfrey-Harris	2023	5	A large amount and depth of qualitative data that relate in depth to the synthesis objective.
Goffer	2022	4	A good amount and depth of qualitative data that relate to the synthesis objective.
Golson	2023	N/A	Maximum variation – Population.
Golson	2022	N/A	Maximum variation – Population.

Gronneberg	2024	5	A large amount and depth of qualitative data that relate in depth to the synthesis objective.
Gudka	2024	N/A	Maximum variation – Dimension of lived experience.
Janssens	2020	4	A good amount and depth of qualitative data that relate to the synthesis objective.
Kwon	2018	N/A	Maximum variation – Country.
Lasky	2016	5	A large amount and depth of qualitative data that relate in depth to the synthesis objective.
Lee	2014	N/A	Maximum variation – Dimension of lived experience.
Loe	2008	5	A large amount and depth of qualitative data that relate in depth to the synthesis objective.
Lyhne	2021	4	A good amount and depth of qualitative data that relate to the synthesis objective.
Meaux	2009	5	A large amount and depth of qualitative data that relate in depth to the synthesis objective.
Oster	2020	5	A large amount and depth of qualitative data that relate in depth to the synthesis objective.
Perry	2006	4	A good amount and depth of qualitative data that relate to the synthesis objective.
Rasmussen	2024	5	A large amount and depth of qualitative data that relate in depth to the synthesis objective.
Schaefer	2017	3	A reasonable amount of qualitative data that relate to the synthesis objective.
Sibley	2018	3	A reasonable amount of qualitative data that relate to the synthesis objective.
Stamp	2014	5	A large amount and depth of qualitative data that relate in depth to the synthesis objective.
Tov	2022	N/A	Maximum variation – Population (age & area).
Weisner	2018	3	A reasonable amount of qualitative data that relate to the synthesis objective
Wiener	2016	5	A large amount and depth of qualitative data that relate in depth to the synthesis objective.

References

Ben-Dor Cohen M, Nahum M, Traub Bar-Ilan R, Eldar E, Maeir A. Coping with emotional dysregulation among young adults with adhd: A mixed-method study of self-awareness and strategies in daily life. Neuropsychological Rehabilitation. 2023 Nov 16.

Brinkman WB, Sherman SN, Zmitrovich AR, Visscher MO, Crosby LE, Phelan KJ, Donovan EF. In their own words: adolescent views on ADHD and their evolving role managing medication. Academic Pediatrics. 2012 Jan 1;12(1):53-61.

Chen W, Epstein A, Toner M, Murphy N, Rudaizky D, Downs J. Enabling successful life engagement in young people with ADHD: New components beyond adult models of recovery. Disability and Rehabilitation. 2023 Jul 3;45(14):2288-300.

Cheung KK, Wong IC, Ip P, Chan PK, Lin CH, Wong LY, Chan EW. Experiences of adolescents and young adults with ADHD in Hong Kong: treatment services and clinical management. BMC Psychiatry. 2015 May 1;15(1):95.

Clancy J, O'Connor J, Ni Mhaolain C. Gripped by the chaos: a psychoanalytically-informed qualitative exploration of adolescent ADHD. Psychoanalytic Psychotherapy. 2020 Jul 2;34(3):228-45.

Darby A. Doctoral Students with LD and/or ADHD: Decision to Disclose and/or Request Accommodations. Learning Disabilities: A Contemporary Journal. 2023;21(2):195-210.

Druedahl LC, Kälvemark Sporrong S. Managing complexity: exploring decision making on medication by young adults with ADHD. Pharmacy. 2018 Apr 19;6(2):33.

Fleishmann A, Kaliski A. Personal autonomy and authenticity: Adolescents' discretionary use of methylphenidate. Neuroethics. 2017 Oct;10(3):419-30.

Godfrey-Harris M, Shaw SC. The experiences of medical students with ADHD: A phenomenological study. PLoS One. 2023 Aug 22;18(8):e0290513.

Goffer A, Cohen M, Maeir A. Occupational experiences of college students with ADHD: A qualitative study. Scandinavian Journal of Occupational Therapy. 2022 Jul 4;29(5):403-14.

Golson ME, McClain MB, Roanhorse TT, Rodríguez MM, Galliher RV. The experience of ADHD as reported by racially and ethnically minoritized adolescents: a survey-based phenomenological investigation. Journal of Racial and Ethnic Health Disparities. 2023 Oct;10(5):2565-76.

Golson ME, Roanhorse TT, McClain MB, Galliher RV, Domenech Rodríguez MM. School-based ADHD services: Perspectives from racially and ethnically minoritized students. Psychology in the Schools. 2022 Apr;59(4):726-43.

Grønneberg SV, Engebretsen E, Løkkeberg ST. Stories of hope: Young people's personal narratives about ADHD put into context of positive aspects. Qualitative Health Research. 2024 Jan;34(1-2):48-60.

Gudka R, Becker K, Ward J, Smith J, Mughal F, Melendez-Torres GJ, Newlove-Delgado T, Price A. Primary care provision for young people with ADHD: a multi-perspective qualitative study. British Journal of General Practice. 2024 Feb 5.

Janssens A, Eke H, Price A, Newlove-Delgado T, Blake S, Ani C, Asherson P, Beresford BA, Emmens T, Hollis C, Logan S. The transition from children's services to adult services for young people with attention deficit hyperactivity disorder: the CATCh-uS mixed-methods study. Health Services and Delivery Research. 2020 Nov 1:1-56.

Kwon SJ, Kim Y, Kwak Y. Difficulties faced by university students with self-reported symptoms of attention-deficit hyperactivity disorder: a qualitative study. Child and Adolescent Psychiatry and Mental Health. 2018 Feb 1;12(1):12.

Lasky AK, Weisner TS, Jensen PS, Hinshaw SP, Hechtman L, Arnold LE, Murray DW, Swanson JM. ADHD in context: Young adults' reports of the impact of occupational environment on the manifestation of ADHD. Social Science & Medicine. 2016 Jul 1;161:160-8.

Lee H, Dunn JC, Holt NL. Youth sport experiences of individuals with attention deficit/hyperactivity disorder. Adapted Physical Activity Quarterly. 2014 Oct 1;31(4):343-61.

Loe M, Cuttino L. Grappling with the medicated self: The case of ADHD college students. Symbolic Interaction. 2008 Jul;31(3):303-23.

Lyhne CN, Pedersen P, Nielsen CV, Bjerrum MB. Needs for occupational assistance among young adults with ADHD to deal with executive impairments and promote occupational participation–a qualitative study. Nordic Journal of Psychiatry. 2021 Jul 1;75(5):362-9.

Meaux JB, Green A, Broussard L. ADHD in the college student: A block in the road. Journal of Psychiatric and Mental Health Nursing. 2009 Apr;16(3):248-56.

Öster C, Ramklint M, Meyer J, Isaksson J. How do adolescents with ADHD perceive and experience stress? An interview study. Nordic Journal of Psychiatry. 2020 Feb 17;74(2):123-30.

Perry SN, Franklin KK. I'm Not the Gingerbread Man! Exploring the Experiences of College Students Diagnosed with ADHD. Journal of Postsecondary Education and Disability. 2006;19(1):94-109.

Rasmussen IL, Schei J, Ørjasæter KB. "A bit lost"—Living with attention deficit hyperactivity disorder in the transition between adolescence and adulthood: an exploratory qualitative study. BMC Psychology. 2024 Jan 11;12(1):20.

Schaefer MR, Rawlinson AR, Wagoner ST, Shapiro SK, Kavookjian J, Gray WN. Adherence to attention-deficit/hyperactivity disorder medication during the transition to college. Journal of Adolescent Health. 2017 Jun 1;60(6):706-13.

Sibley MH, Yeguez CE. Managing ADHD at the post-secondary transition: A qualitative study of parent and young adult perspectives. School Mental Health. 2018 Dec;10(4):352-71.

Stamp L, Banerjee M, Brown FC. Self-Advocacy and Perceptions of College Readiness among Students with ADHD. Journal of Postsecondary Education and Disability. 2014;27(2):139-60.

Siman Tov A, Halevi Hochwald I, Tesler R, Green G. Weight Management for Students with Attention-Deficit Hyperactivity Disorder (ADHD): A Qualitative Study. InHealthcare 2022 Nov 7 (Vol. 10, No. 11, p. 2225). MDPI.

Weisner TS, Murray DW, Jensen PS, Mitchell JT, Swanson JM, Hinshaw SP, Wells K, Hechtman L, Molina BS, Arnold LE, Sorensen P. Follow-up of young adults with ADHD in the MTA: Design and methods for qualitative interviews. Journal of Attention Disorders. 2018 Jul;22(9_suppl):10S-20S.

Wiener J, Daniels L. School experiences of adolescents with attention-deficit/hyperactivity disorder. Journal of Learning Disabilities. 2016 Nov;49(6):567-81.

Papers for Exclusion

Author	Year	Score	Comment

Ahmad	2024	1	Very little qualitative data presented that relate to the synthesis objective.
Denyer	2023	1	Very little qualitative data presented that relate to the synthesis objective.
Druedahl	2020	2	Some qualitative data presented that relate to the synthesis objective.
Enggaard	2020	2	Some qualitative data presented that relate to the synthesis objective.
Frondelius	2019	2	Some qualitative data presented that relate to the synthesis objective.
Gallo	2014	2	Some qualitative data presented that relate to the synthesis objective.
Gallagher	2023	1	Very little qualitative data presented that relate to the synthesis objective.
Gibbs	2016	2	Some qualitative data presented that relate to the synthesis objective.
Gibbs	2022	2	Some qualitative data presented that relate to the synthesis objective.
Gray	2016	1	Those findings that are presented are fairly descriptive.
Hareendran	2015	2	Some qualitative data presented that relate to the synthesis objective.
Lee	2008	1	Very little qualitative data presented that relate to the synthesis objective. Those findings that are presented are fairly descriptive.
Meaux	2006	2	Some qualitative data presented that relate to the synthesis objective.
Meyer	2020	1	Very little qualitative data presented that relate to the synthesis objective.
Mitchell	2018	1	Those findings that are presented are fairly descriptive.
Padilla-Petry	2018	2	Some qualitative data presented that relate to the synthesis objective.
Parker	2011	1	Very little qualitative data presented that relate to the synthesis objective.
Parker	2013	1	Very little qualitative data presented that relate to the synthesis objective.
Pfeifer	2023	1	Very little qualitative data presented that relate to the synthesis objective.
Sibley	2023	1	Very little qualitative data presented that relate to the synthesis objective. Those findings that are presented are fairly descriptive.
Sikirica	2015	2	Some qualitative data presented that relate to the synthesis objective.
Taneja-Johansson	2021	2	Some qualitative data presented that relate to the synthesis objective.
Wong	2009	2	Some qualitative data presented that relate to the synthesis objective.
Yeguez	2022	1	Very little qualitative data presented that relate to the synthesis objective. Those findings that are presented are fairly descriptive.
Zaghi	2023	1	Very little qualitative data presented that relate to the synthesis objective.

References

Ahmad SI, Hinshaw SP. A qualitative analysis of perspectives on self-directed violence in a prospective longitudinal study of young women with and without childhood ADHD. Journal of Attention Disorders. 2024 Jul;28(9):1255-66.

Denyer H, Deng Q, Adanijo A, Asherson P, Bilbow A, Folarin A, Groom MJ, Hollis C, Wykes T, Dobson RJ, Kuntsi J. Barriers to and facilitators of using remote measurement technology in the long-term monitoring of individuals with ADHD: interview study. JMIR Formative Research. 2023 Jun 30;7(1):e44126.

Druedahl LC, Sporrong SK. More than meets the eye: A Foucauldian perspective on treating ADHD with medicine. Research in Social and Administrative Pharmacy. 2020 Sep 1;16(9):1201-7.

Enggaard H, Laugesen B, Zoffmann V, Lauritsen MB, Jørgensen R. Adolescents' Perceptions of Living With Co-Existing ADHD and Medical Disorder in Denmark. Journal of Pediatric Nursing. 2020 Jul 1;53:e129-35.

Frondelius IA, Ranjbar V, Danielsson L. Adolescents' experiences of being diagnosed with attention deficit hyperactivity disorder: a phenomenological study conducted in Sweden. BMJ open. 2019 Aug 1;9(8):e031570.

Gallagher J, McDonnell M. JournalMate: An Accessible Academic Reading Tool for Third-Level Students with ADHD. InAssistive Technology: Shaping a Sustainable and Inclusive World 2023 (pp. 207-214). IOS Press.

Gallo MP, Mahar P, Chalmers L. College student's perceptions of living and learning with attention deficit hyperactivity disorder (ADHD). The Journal of Special Education Apprenticeship. 2014;3(2):2.

Gibbs K, Mercer KL, Carrington S. The schooling experience of adolescent boys with AD/HD: An Australian case study. International Journal of Disability, Development and Education. 2016 Nov 1;63(6):608-22.

Gibbs K, Carrington S, Mercer KL. Perspectives about friendships and the school learning environment from Australian adolescent boys with AD/HD. International Journal of Disability, Development and Education. 2022 Nov 2;69(6):1974-87.

Gray SA, Fettes P, Woltering S, Mawjee K, Tannock R. Symptom manifestation and impairments in college students with ADHD. Journal of Learning Disabilities. 2016 Nov;49(6):616-30.

Hareendran A, Setyawan J, Pokrzywinski R, Steenrod A, Madhoo M, Erder MH. Evaluating functional outcomes in adolescents with attention-deficit/hyperactivity disorder: development and initial testing of a self-report instrument. Health and Quality of Life Outcomes. 2015 Aug 22;13(1):133.

Lee KS, Osborne RE, Hayes KA, Simoes RA. The effects of pacing on the academic testing performance of college students with ADHD: A mixed methods study. Journal of Educational Computing Research. 2008 Sep;39(2):123-41.

Meaux JB, Hester C, Smith B, Shoptaw A. Stimulant medications: A trade-off? The lived experience of adolescents with ADHD. Journal for Specialists in Pediatric Nursing. 2006 Oct;11(4):214-26.

Meyer J, Öster C, Ramklint M, Isaksson J. You are not alone–adolescents' experiences of participation in a structured skills training group for ADHD. Scandinavian Journal of Psychology. 2020 Oct;61(5):671-8.

Mitchell JT, Weisner TS, Jensen PS, Murray DW, Molina BS, Arnold LE, Hechtman L, Swanson JM, Hinshaw SP, Victor EC, Kollins SH. How substance users with ADHD perceive the relationship between substance use and emotional functioning. Journal of Attention Disorders. 2018 Jul;22(9_suppl):49S-60S.

Padilla-Petry P, Sòria-Albert C, Vadeboncoeur JA. Experiencing Disability in the School Context: Voices of Young People Diagnosed with ADHD in Spain. International Education Studies. 2018;11(8):79-90.

Parker DR, Hoffman SF, Sawilowsky S, Rolands L. An Examination of the Effects of ADHD Coaching on University Students' Executive Functioning. Journal of Postsecondary Education and Disability. 2011;24(2):115-32.

Parker DR, Hoffman SF, Sawilowsky S, Rolands L. Self-control in postsecondary settings: Students' perceptions of ADHD college coaching. Journal of Attention Disorders. 2013 Apr;17(3):215-32.

Pfeifer MA, Cordero JJ, Stanton JD. What I wish my instructor knew: How active learning influences the classroom experiences and self-advocacy of STEM majors with ADHD and specific learning disabilities. CBE—Life Sciences Education. 2023;22(1):ar2.

Sibley MH, Shelton CR, Garcia I, Monroy JM, Hill DM, Johansson M, Link K, Greenwood L, Torres Antunez G, Reyes Francisco JC. Are there long-term effects of behavior therapy for adolescent ADHD? A qualitative study. Child Psychiatry & Human Development. 2023 Aug;54(4):985-96.

Sikirica V, Flood E, Dietrich CN, Quintero J, Harpin V, Hodgkins P, Skrodzki K, Beusterien K, Erder MH. Unmet needs associated with attention-deficit/hyperactivity disorder in eight European countries as reported by caregivers and adolescents: results from qualitative research. The Patient-Patient-Centered Outcomes Research. 2015 Jun;8(3):269-81.

Taneja-Johansson S. Turning points in the educational pathways of young people with attention-deficit/hyperactivity disorder in Sweden. International Journal of Inclusive Education. 2021.

Wong IC, Asherson P, Bilbow A, Clifford S, Coghill D, DeSoysa R, Hollis C, McCarthy S, Murray M, Planner C, Potts L. Cessation of attention deficit hyperactivity disorder drugs in the young (CADDY)-pharmacoepidemiological and qualitative study. Health Technology Assessment. 2009;13(50):1-44.

Yeguez CE, Ogle RR, Jusko ML, Melendez R, Sibley MH. The impact of an intensive summer treatment program for adolescents with ADHD: A qualitative study of parent and young adult perspectives. Journal of Child and Family Studies. 2022 Dec;31(12):3281-301.

Zaghi AE, Grey A, Hain A, Syharat CM. "It Seems Like I'm Doing Something More Important"—An Interpretative Phenomenological Analysis of the Transformative Impact of Research Experiences for STEM Students with ADHD. Education Sciences. 2023 Jul 28;13(8):776.
