# Supplementary material for: “It has tentacles into every single aspect of me” a qualitative evidence synthesis of the lived experiences and perceptions of ADHD youth
Source: Eur Child Adolesc Psychiatry. 2026 Feb 25;35(5):1435–49. doi: 10.1007/s00787-025-02955-8 (PMC13272611; doi:10.1007/s00787-025-02955-8)
Supplement: Supplementary file 4 — (PDF 165 KB) [file 787_2025_2955_MOESM4_ESM.pdf]

"It has tentacles into every single aspect of me" A Qualitative Evidence Synthesis of the Lived Experiences and Perceptions of ADHD Youth.

European Child & Adolescent Psychiatry

Jessie Tierney[1], Health Research Institute, School of Allied Health, Faculty of Education and Health Sciences, University of Limerick, Ireland. tierney.jessie@ul.ie

Doctor Ann-Marie Morrissey[2], Ageing Research Centre, Health Research Institute, School of Allied Health, Faculty of Education and Health Sciences, University of Limerick, Ireland.

Doctor Dimitrios Adamis[3], Sligo Mental Health Services Adult ADHD Clinic, Sligo, Ireland; and Department of Psychiatry, University of Limerick, Ireland.

Doctor Margo Wrigley[4], HSE National Clinical Programme for ADHD in Adults, Health Service Executive, Dublin 8, Ireland.

Doctor Katie Robinson[2], Ageing Research Centre, Health Research Institute, School of Allied Health, Faculty of Education and Health Sciences, University of Limerick, Ireland.

The eMERGe meta-ethnography reporting guidance

France EF, Cunningham M, Ring N, Uny I, Duncan EA, Jepson RG, Maxwell M, Roberts RJ, Turley RL, Booth A, Britten N. Improving reporting of meta-ethnography: the eMERGe reporting guidance. BMC Medical Research Methodology. 2019 Jan 31;19(1):25.

No.	Criteria Headings	Reporting Criteria
Phase 1—Selecting meta-ethnography and getting started		
Introduction		
1	Rationale and context for the meta-ethnography	Describe the gap in research or knowledge to be filled by the meta-ethnography, and the wider context of the meta-ethnography<br><br>Page 3 - 4
2	Aim(s) of the meta-ethnography	Describe the meta-ethnography aim(s)<br><br>Page 4
3	Focus of the meta-ethnography	Describe the meta-ethnography review question(s) (or objectives)<br><br>Page 4
4	Rationale for using meta-ethnography	Explain why meta-ethnography was considered the most appropriate qualitative synthesis methodology<br>Page 4
Phase 2—Deciding what is relevant		
Methods		

5	Search strategy	Describe the rationale for the literature search strategy<br>Page 4 - 5
6	Search processes	Describe how the literature searching was carried out and by whom<br>Page 4 - 5
7	Selecting primary studies	Describe the process of study screening and selection, and who was involved<br><br>Page 5 - 6
Findings		
8	Outcome of study selection	Describe the results of study searches and screening<br><br>Page 7
Phase 3—Reading included studies		
Methods		
9	Reading and data extraction approach	Describe the reading and data extraction method and processes<br><br>Page 6
Findings		
10	Presenting characteristics of included studies	Describe characteristics of the included studies<br><br>Page 7
Phase 4—Determining how studies are related		
Methods		
11	Process for determining how studies are related	Describe the methods and processes for determining how the included studies are related:<br><br>- Which aspects of studies were compared<br><br>AND<br><br>- How the studies were compared<br><br>Page 6
Findings		
12	Outcome of relating studies	Describe how studies relate to each other<br><br>Page 8 - 13

Phase 5—Translating studies into one another		
Methods		
13	Process of translating studies	Describe the methods of translation**:**<br><br>- Describe steps taken to preserve the context and meaning of the relationships between concepts within and across studies- Describe how the reciprocal and refutational translations were conducted- Describe how potential alternative interpretations or explanations were considered in the translations<br><br>Page 6
Findings		
14	Outcome of translation	Describe the interpretive findings of the translation.<br>Page 8 - 13
Phase 6—Synthesizing translations		
Methods		
15	Synthesis process	Describe the methods used to develop overarching concepts (“synthesised translations”)Describe how potential alternative interpretations or explanations were considered in the synthesis<br>Page 6
Findings		
16	Outcome of synthesis process	Describe the new theory, conceptual framework, model, configuration, or interpretation of data developed from the synthesis<br>Page 13 - 16
Phase 7—Expressing the synthesis		
Discussion		
17	Summary of findings	Summarize the main interpretive findings of the translation and synthesis and compare them to existing literature<br><br>Page 13 - 16
18	Strengths, limitations, and reflexivity	Reflect on and describe the strengths and limitations of the synthesis:<br><br>- Methodological aspects—for example, describe how the synthesis findings were influenced by the nature of the included studies and how the meta-ethnography was conducted.- Reflexivity—for example, the impact of the research team on the synthesis findings<br><br>Page 4 & 16

19	Recommendations and conclusions	Describe the implications of the synthesis<br><br>Page 16
