# Supplementary material for: “It has tentacles into every single aspect of me” a qualitative evidence synthesis of the lived experiences and perceptions of ADHD youth
Source: Eur Child Adolesc Psychiatry. 2026 Feb 25;35(5):1435–49. doi: 10.1007/s00787-025-02955-8 (PMC13272611; doi:10.1007/s00787-025-02955-8)
Supplement: Supplementary file 5 — (PDF 242 KB) [file 787_2025_2955_MOESM5_ESM.pdf]

“It has tentacles into every single aspect of me” A Qualitative Evidence Synthesis of the Lived Experiences and Perceptions of ADHD Youth.

European Child & Adolescent Psychiatry

Jessie Tierney[1], Health Research Institute, School of Allied Health, Faculty of Education and Health Sciences, University of Limerick, Ireland. tierney.jessie@ul.ie

Doctor Ann-Marie Morrissey[2], Ageing Research Centre, Health Research Institute, School of Allied Health, Faculty of Education and Health Sciences, University of Limerick, Ireland.

Doctor Dimitrios Adamis[3], Sligo Mental Health Services Adult ADHD Clinic, Sligo, Ireland; and Department of Psychiatry, University of Limerick, Ireland.

Doctor Margo Wrigley[4], HSE National Clinical Programme for ADHD in Adults, Health Service Executive, Dublin 8, Ireland.

Doctor Katie Robinson[2], Ageing Research Centre, Health Research Institute, School of Allied Health, Faculty of Education and Health Sciences, University of Limerick, Ireland.

CASP Summary Table

Author	Year	Q.1. Was there a clear statement of the aims of the research?	Q.2. Is a qualitative methodology appropriate?	Q.3. Was the research design appropriate to address the aims of the research?	Q.4. Was the research design appropriate to address the aims of the research?	Q.5. Was the data collected in a way that addressed the research issue?	Q.6. Has the relationship between research and participants been adequately considered?	Q.7. Have ethical issues been taken into consideration?	Q.8. Was the data analysis sufficiently rigorous?	Q.9. Is there a clear statement of findings?	Q.10. How valuable is the research?
Ben-Dor Cohen	2023	Yes	Yes	Yes	Can’t tell	No	No	Yes	Yes	Yes	Yes
Brinkman	2012	Yes	Yes	Can’t tell	Yes	Can’t tell	No	Yes	Yes	Can’t tell	Yes
Chen	2023	Yes	Yes	Yes	Yes	Yes	No	Yes	Yes	Yes	Yes
Cheung	2015	Yes	Yes	Yes	Yes	Can’t tell	No	Yes	Yes	Yes	Yes
Clancy	2020	Yes	Yes	Yes	Yes	Yes	Can’t tell	Yes	Can’t tell	Yes	Yes
Darby	2023	Yes	Yes	Yes	Yes	Yes	Yes	No	Yes	Yes	Yes
Druedahl	2018	Yes	Yes	Yes	Yes	Can’t tell	No	Yes	Can’t tell	Yes	Yes
Fleishmann	2017	Yes	Yes	Yes	Can’t tell	Yes	No	Yes	Yes	Yes	Yes

Godfrey-Harris	2023	Yes	Yes	Yes	Yes	Yes	Can’t Tell	Yes	Yes	Yes	Yes
Goffer	2022	Yes	Yes	Yes	Can’t tell	Yes	No	Yes	Yes	Yes	Yes
Golson	2023	Yes	Yes	Yes	Yes	Yes	Yes	Yes	Can’t tell	Yes	Yes
Golson	2022	Yes	Yes	Yes	Yes	Yes	Yes	Yes	Can’t tell	Yes	Yes
Gronneberg	2024	Yes	Yes	Yes	Yes	Yes	No	Yes	Yes	Yes	Yes
Gudka	2024	Yes	Yes	Yes	Yes	Yes	No	Yes	Yes	Yes	Yes
Janssens	2020	Yes	Yes	Yes	Yes	Yes	No	Yes	Yes	Yes	Yes
Kwon	2018	Yes	Yes	Yes	Yes	Yes	No	Yes	Yes	Yes	Yes
Lasky	2016	Yes	Yes	Yes	Yes	Yes	No	Yes	Yes	Yes	Yes
Lee	2014	Yes	Yes	Yes	Yes	Yes	Yes	Yes	Yes	Yes	Yes
Loe	2008	Yes	Yes	Yes	Yes	Yes	No	No	Can’t tell	Yes	Yes
Lyhne	2021	Yes	Yes	Yes	Yes	Yes	No	Yes	Yes	Yes	Yes
Meaux	2009	Yes	Yes	Yes	Yes	Yes	No	Yes	Yes	Yes	Yes
Oster	2020	Yes	Yes	Yes	Yes	Yes	Yes	Yes	Yes	Yes	Yes
Perry	2006	Yes	Yes	Yes	Yes	Yes	No	No	Yes	Yes	Yes
Rasmussen	2024	Yes	Yes	Yes	Yes	Yes	Yes	Yes	Yes	Yes	Yes
Schaefer	2017	Yes	Yes	Yes	Yes	Yes	No	Yes	Yes	Yes	Yes
Sibley	2018	Yes	Yes	Yes	Yes	Yes	Yes	Yes	Yes	Yes	Yes
Stamp	2014	Yes	Yes	Yes	Yes	Yes	No	No	Yes	Yes	Yes
Tov	2022	Yes	Yes	Yes	Yes	Can’t tell	No	Yes	Yes	Yes	Yes
Weisner	2018	Yes	Yes	Yes	Yes	Yes	No	Yes	Yes	Yes	Yes
Wiener	2016	Yes	Yes	Yes	Yes	Yes	No	No	Yes	Yes	Yes
