# Supplementary material for: “It has tentacles into every single aspect of me” a qualitative evidence synthesis of the lived experiences and perceptions of ADHD youth
Source: Eur Child Adolesc Psychiatry. 2026 Feb 25;35(5):1435–49. doi: 10.1007/s00787-025-02955-8 (PMC13272611; doi:10.1007/s00787-025-02955-8)
Supplement: Supplementary file 6 — (PDF 242 KB) [file 787_2025_2955_MOESM6_ESM.pdf]

"It has tentacles into every single aspect of me" A Qualitative Evidence Synthesis of the Lived Experiences and Perceptions of ADHD Youth.

European Child & Adolescent Psychiatry

Jessie Tierney[1], Health Research Institute, School of Allied Health, Faculty of Education and Health Sciences, University of Limerick, Ireland. tierney.jessie@ul.ie

Doctor Ann-Marie Morrissey[2], Ageing Research Centre, Health Research Institute, School of Allied Health, Faculty of Education and Health Sciences, University of Limerick, Ireland.

Doctor Dimitrios Adamis[3], Sligo Mental Health Services Adult ADHD Clinic, Sligo, Ireland; and Department of Psychiatry, University of Limerick, Ireland.

Doctor Margo Wrigley[4], HSE National Clinical Programme for ADHD in Adults, Health Service Executive, Dublin 8, Ireland.

Doctor Katie Robinson[2], Ageing Research Centre, Health Research Institute, School of Allied Health, Faculty of Education and Health Sciences, University of Limerick, Ireland.

Table of Characteristics of Included Studies

Legend:
W = Women
M = Men
Y.O. = Years old

Author	Year	Country	Recruitment Setting	Aim	Sample	Diagnosis	Data Collection	Method	ADHD Medication Use	Treatments Engaged in	Findings
Ben-Dor Cohen	2023	Israel	Online advertisement	To gain better understanding of self-awareness of ED and regulatory strategies in the daily life of young adults with ADHD	n = 60<br><br>32W & 28M<br><br>18-33Y.O.	Formal diagnosis n = 60	Self-Regulation Skills Interview (Semi-structured)	Mixed method with thematic analysis combined with a directed approach	n = 41 reported no medication use, n = 14 reported occasional use, and n =5 reported daily use.	Not reported	Significant challenges in self-awareness and strategies were demonstrated qualitatively. Qualitative content analysis revealed a range of self-awareness levels, which were related to noticing ED-related cues and understanding contextual factors predictive of ED. Self-awareness and strategies were significantly associated. Strategies varied regarding effort, individual preference and temporality.
Brinkman	2012	United States	Community-based pediatric practices	Understanding of how adolescents with ADHD contribute to	n = 44<br><br>20W & 24M	Formal diagnosis n = 44	Focus groups	Qualitative research methods with an inductive	Medication trial during childhood was reported by 97%.	Not reported	Adolescents assumed increased responsibility for managing medication as they matured and developed insight into the functional impact of ADHD

				medication treatment decisions	13-18Y.O.			approach to coding	Continued medication use as adolescents was reported by 68%		and medication on their lives. Insights were often formed by contrasting time spent on and off medication. ADHD impacted functioning in the following domains: academics, social interactions and relationships, creativity, and driving skills. Select domains were relevant for some adolescents but not others. Adolescents described different roles that they played in managing medication as well as strategies they used to exert autonomy over medication use. Side effects were common and contributed to negative feelings toward medication. Some adolescents had begun to use medication selectively. Many expressed uncertainty about future use of medication.
Chen	2023	Australia	ADHD organisation	To examine the lived experiences of young people successfully managing life with ADHD and investigate the applicability of adult models of Recovery to these individuals	n = 27<br><br>11W & 16M<br><br>15-31Y.O.	Formal diagnosis n = 27	Semi-structured interview	Modified grounded theory methodology with thematic analysis	Number of participants taking medication not explicitly stated.	No overarching description of non-pharmacological therapy use; individual findings note that some participants consulted coaches, psychologists and psychiatrists	The emergent framework comprised four elements: (i) Recovernance (RE) (a portmanteau merging 'Recovery' and 'Maintenance'; ongoing adjustment to maintain one's personal best without an end point); (ii) Personal Optimization (PO) (continuously striving to maximize function and adjust one's goals given fluctuating impairments and internal resources); (iii) Self (S) (facing internal challenges and developing internal resilience); and (iv) Environment (E) (facing external challenges and fostering external resilience). These four elements yielded the acronym 'REPOSE'.
Cheung	2015	Hong Kong	University pediatrics and adolescent medicine department & child and	Explore patients' experiences and opinions towards ADHD	n = 40<br><br>13W & 27M	Formal diagnosis n = 40	Semi-structured interview	Qualitative research methods with a thematic approach	Using medication n = 33	ADHD training/Behavioural therapy n = 18	Four meta-themes were developed: Accessing ADHD diagnosis and treatment services; ADHD-related impairment; Experience of ADHD treatments; and

			adolescent psychiatric hospital team	treatment in Hong Kong	16-23Y.O.			to analysis based on grounded theory	Discontinued medication n = 7	Psychological Counselling n = 3<br><br>Both of the above treatments n = 2	Attitudes and expectations of future ADHD treatment. The role of parents and schools were highly significant in accessing services for patients diagnosed with ADHD in childhood. In general, ADHD affected every aspect of patients' lives including academic outcome, employment, family and social relationships. Medications were the principal treatment for ADHD amongst the interviewees and were reported to be generally effective. Half of the patients received non-pharmacological treatments in childhood but these effects were reported to be temporary. There was general consensus that the needs of patients with ADHD could not be met by the current service. In particular, there is a lack of specialist service for adults with ADHD, follow-up by different clinicians, and insufficient provision of non-pharmacological treatments.
Clancy	2020	Ireland	Child and adolescent mental health service	Explore adolescents' experiences of ADHD through a psychoanalytic lens, with the aim of deepening our understanding from an interpersonal and emotional standpoint	n = 5<br><br>4W & 1M<br><br>16-17Y.O.	Formal diagnosis n = 5	Semi-structured interview	Qualitative study with psychoanalytically informed method of analysis	Using medication n = 4<br><br>Discontinued medication n = 1	Engaged in Child and Adolescent Mental Health Services (CAMHS) n = 5	Three inter-related themes emerged: 'not being able to "let out" difficult emotion,' which reflected participants' struggle with overwhelming and unmanageable affect; '(un)soothing experience with self and other,' which described the relationship between care-seeking and care-giving behaviours in the lives of these youths; and 'being in and out of control,' which relates to the complex relationship that participants have with self-control as well as the control of others
Darby	2023	United States	Universities	To examine the factors associated	n = 19	Formal diagnosis n = 19	Semi-structured interview	Constructivist theory with cycle	Using medication n = 15	No overarching description of non-	Findings showed that doctoral students with LD and/or ADHD decide on disclosing their

				with doctoral students with learning disabilities (LD) and/or attention-deficit/hyperactivity disorder (ADHD) disclosing to the university disability resources office, their instructors, and their faculty advisors	13W & 6M<br><br>age range not reported			of coding analysis		pharmacological therapy use; individual findings note that some participants accessed university disability services	disability and requesting an accommodation when the need outweighs the risk. Doctoral students with LD and/or ADHD disclosed when they needed accommodations in their courses, on comprehensive exams, or for writing research papers and/or their dissertation. Seven students disclosed to their disability resources office to request accommodations in their coursework. The areas in which participants needed the most support were writing research papers and their dissertation. Eighteen of the 21 participants went to their advisor for this support. Those who did not disclose made this choice due to not needing support or fear of how they would be perceived.
Druedahl	2018	Denmark	ADHD patient association	Explore the beliefs and experiences of young adults with ADHD related to their medication treatment decisions	n = 10<br><br>7W & 3M<br><br>22-29Y.O.	Formal diagnosis n = 10	Semi-structured interview & Focus groups	Qualitative methods with conventional content analysis	Using medication n = 7<br><br>Discontinued use n = 3	No overarching description of non-pharmacological therapy use; individual findings note that some participated in psychoeducation and ADHD groups	Three major themes were identified: (1) the patient's right to choose concerning ADHD medicine; (2) the patient's decision of whether or not to treat ADHD with medication; and (3) factors affecting the patient's decision on whether to take ADHD medication or not. The latter theme contained 15 factors, which were distributed across three levels: individual, between-individuals, and societal. The dominant factors were increasing quality of life and improving oneself e.g., improving social skills.
Fleishmann	2017	Israel	Research assistants selected participants whom they personally knew had ADHD	To understand how psychological changes that young people experience when they take these	n = 38<br><br>20W & 18M<br><br>15-19Y.O.	Formal diagnosis n = 38	Semi-structured interview	Qualitative study with grounded theory analysis	All participants were taking methylphenidate regularly or intermittently or took it previously for a period of more than a year	Not reported	The findings, analyzed using grounded theory, show that methylphenidate affects the participants' demeanor, mood, and even preferences. The participants, aware of these effects, apply discretion in taking methylphenidate and thus influence their traits and their willingness to engage in

				medications interrelate with their attitude toward being medicated							various activities. When needing to prepare for a matriculation exam, for example, they take methylphenidate; when they need to be creative or sociable, they avoid it and enjoy what they consider the advantages of ADHD, such as creativity and spontaneity. As discretionary users, they shape their life stories in a way that makes them more meaningful and diverse, better tailored to their social surroundings, and more useful in maintaining personal autonomy in the course of pharmacological treatment of ADHD.
Godfrey-Harris	2023	England	Medical School	What are the experiences of medical students with ADHD	n = 6<br><br>4F, 1M & 1Non-Binary<br><br>20-25Y.O.	Formal diagnosis n = 6	Semi-structured interview	Qualitative study with interpretive phenomenological approach	Number of participants taking medication not explicitly stated.	Not reported	Our analysis identified the following themes: Identity and diagnosis; ADHD profile; system issues; conflict, competition and compensation; improving the experience. Participants reported experiences of bullying and isolation at medical school, perpetrated by doctors and peers, as well as feelings of alienation when unable to conform on placement and in exams. From this, participants adopted survival strategies, such as masking, to avoid being ostracised. All recognised their ADHD status when their mental health deteriorated during their medical studies. Of those who disclosed their diagnosis, none were offered personalised support. Participants feared disclosure, largely due to weaponised professionalism and the effects of toxic competitiveness in medicine. They yearned for a sense of belonging. Participants reported strengths associated with ADHD such as empathy and

											working well under pressure, which are highly desirable aptitudes for doctors.
Goffer	2022	Israel	Colleges	Gaining a deeper understanding of the occupational experiences of college students with ADHD and exploring factors that facilitate or impede their occupational performance	n = 20<br><br>11W & 9M<br><br>20-32Y.O.	Formal diagnosis n = 20	Semi-structured interview	Qualitative study with qualitative content analysis	Number of participants taking medication not explicitly stated.	Not reported	Six themes were found in relation to varied occupational domains: (1) Eating and meal preparation: Too little or too much; (2) Sleep: Not enough to 'recharge batteries'; (3) Medication management: Intense ambivalence; (4) Studying: Too hard, too effortful (5) Work: Need it and love it; and (6) Leisure: Desired yet absent. Each theme contained categories related to factors that influenced performance. Impeding factors included occupational demands, ADHD biological attributes, and personal beliefs. Facilitating factors included self-awareness, executive strategies, adaptive routines, and enabling social context.
Golson	2023	United States	Online (Qualtrics panel)	The present research was guided by the central question: how do REM adolescents experience ADHD?	n = 35<br><br>14W, 20M & 1Prefer not to say<br><br>14-17Y.O.	Formal diagnosis n = 26	Survey with phenomenological questions	Phenomenological study with transcendental phenomenological data analysis	Number of participants taking medication not explicitly stated.	Not reported	Participant responses to an online phenomenological survey yielded four themes and eleven subthemes related to ADHD symptom experiences, navigating social relationships and school, stigma, and finding positivity in ADHD. Participant experiences coroborate and expand on extant research regarding ADHD symptoms, social isolation, school discipline referrals, and stigma. These findings highlight the importance of including REM participants in research and increased efforts to decrease stigma.
Golson	2022	United States	Online (Qualtrics panel)	To investigate the experiences of REM students	n = 35<br><br>20M	Formal diagnosis n = 26	Survey with phenomenological questions	Phenomenological study with transcendental phenomen	Not reported	No overarching description of non-pharmacological therapy use; individual	Results from this phenomenological investigation revealed 5 themes and 14 subthemes describing REM students' thoughts, feelings, and experiences of ADHD

				receiving school-based ADHD services	Average age 15.8 Y.O.			ological data analysis		findings note that some participants consulted therapists, neuropsycholog ists, or behavioral psychiatrists.	assessment, intervention, and parent involvement in these processes. These findings suggest REM adolescent students should be informed decision-makers in their school-based services, though they are often left out of the planning process. Additionally, student reports support the involvement of parents and interdisciplinary community professionals in school-based services.
Gronnebe rg	2024	Norway	High schools and colleges	This article contributes to research on the positive aspects of the diagnosis, specifically understandin g the positive aspects of living with ADHD.	n = 10<br><br>5W & 5M<br><br>18-28Y.O.	Formal diagnosis n = 10	Semi-structured interview	Qualitativ e study with narrative and discourse analysis	Using medication n = 5<br><br>Occasional use n = 3<br><br>Discontinued use n = 2	All female participants and one male had sought, initiated, or continued therapeutic counselling with a professional therapist; cognitive therapy was also reported	The findings showed that challenges with the diagnosis were not necessarily stably occupied, and for some, the diagnosis was thought of as a benefit and something they would not have been without. Four stories highlighted particularly the context of positive aspects: (1) insight and strategies, (2) targeted efforts, (3) balanced energy, and (4) social skills. These aspects were correlated to both the individual's strengths as well as the strengths and support that could be related to their societal and cultural environment.
Gudka	2024	England	Primary care	To investigate the experiences of young people with ADHD accessing primary care in England	n = 6<br><br>4W & 2M<br><br>17-24Y.O.	Formal diagnosis n= 6	Semi-structured interview	Qualitativ e study with reflexive thematic analysis	Number of participants taking medication not explicitly stated.	All participants engaged in a primary care service	Three themes were generated: a system under stress, incompatibility between ADHD and the healthcare system, and strategies for change.
Janssens	2020	United Kingdom	NHS trusts	To explore how stakeholders experience transition, to identify factors that influence the quality and	n = 64<br><br>20W & 44M<br><br>14-29Y.O.	Formal diagnosis n = 64	Semi-structured interview	Mixed-method design with a qualitative interview study with PPI and framewor	Number of participants taking medication not explicitly stated.	No overarching description of non-pharmacologica l therapy use; individual findings note that some participants	The interviews with patients revealed a lack of understanding of attention deficit hyperactivity disorder, and this particularly related to impairment in adulthood. Patients often associated medication with education and assumed that treatment would

				experience of the process of transition from children's services to AMHS, to identify factors or processes that underlie continuing or discontinuing treatment when approaching the service age boundary.				k analysis approach		sought out interventions such as cognitive–behavioural therapy, group or individual counselling, animal therapy, restricted diets (i.e. avoiding certain foods), exercise or meditation	end when their schooling ended. The medication focus of services meant that those who did stop medication before transition did not transfer to adult services. Those going on to higher education were more likely to transition but still expressed a view that they would stop their medication once they had finished university. Those who did not transition, but after a period without routine care returned to services as a young adult, were often prompted to seek help after a profoundly negative event in their lives, which emphasised the ongoing influence of attention deficit hyperactivity disorder. Parents were more likely to view attention deficit hyperactivity disorder as an impairment that needed ongoing support prior to transition and their active involvement was viewed by all stakeholders as essential for transition to be successful. How prepared a patient and parent were for transition, the quality of patient information handover, accessibility of adult services and the fit of patient needs with the remit of adult services available were all interlinked factors influencing the success of transition. With comorbidities frequently observed in patients with attention deficit hyperactivity disorder, transition often depended on coexisting conditions and the complexity of patient needs.
Kwon	2018	Korea	University	To investigate the difficulties experienced	n = 12<br><br>7W & 5M	Formal diagnosis n = 2	Semi-structured interview	Qualitative study with thematic analysis	Discontinued use n = 1	Counselling n = 1	Difficulties in university life were classified into four main themes (lack of daily routine, unsatisfactory academic performance and achievement,

				by Korean university students with ADHD symptoms so as to provide the basic data needed to develop appropriate interventions	20-29Y.O.						reduced interpersonal skills, and continuing worries) and analyzed. University students with ADHD symptoms had difficulties coping with repeated cycles of negative thoughts and worries, irregu-lar lifestyles due to poor time management, dissatisfaction with academic performance and interpersonal relationships, self-dissatisfaction, and decreased self-esteem.
Lasky	2016	North America	Universities, a children's hospital, university medical center	Do the new contexts young adults find themselves in alter their experience of ADHD? Are there particular occupational or educational contexts in which young adults report functioning better than others?	n = 125<br><br>30W & 95M<br><br>23.3-25.5Y.O.	Formal diagnosis n = 125	Semi-structured interview	Qualitative add-on study with thematic analysis	Number of participants taking medication not explicitly stated.	Not reported	Many subjects describe their symptoms as context-dependent. In some contexts, participants report feeling better able to focus; in others, their symptoms such as high energy levels become strengths rather than liabilities. Modal descriptions included tasks that were stressful and challenging, novel and required multitasking, busy and fast-paced, physically demanding or hands-on, and/or intrinsically interesting. Consistent with a developmental psychopathology framework, ADHD is experienced as arising from an interaction between our subjects and their environments. These findings demonstrate the need to account for the role of context in our understanding of ADHD as a psychiatric disorder, especially as it manifests in young adulthood.
Lee	2014	Canada	University & ADHD association	Explore youth sport experiences of individuals with attention deficit/hyperactivity disorder	n = 6<br><br>0W & 6M<br><br>Mean age 22.7Y.O.	Formal diagnosis n = 6	Semi-structured interview	Interpretive phenomenological analysis methodology and idiographic analysis	Number of participants taking medication not explicitly stated.	Not reported	Findings showed that symptoms of ADHD hampered participants' experiences and led to negative interpersonal and performance-related consequences. On the other hand, participants reported social and stress/energy-release benefits arising from their experiences in sport. Their

											experiences were therefore complex, and some findings relating to social interactions appeared contradictory (e.g., negative interpersonal experiences vs. social benefits). Supportive coaches, understanding teammates, and personal coping strategies were key factors that enabled participants to realize benefits and, to some degree, mitigate negative consequences associated with their participation in sport.
Loe	2008	United States	College	To understand how college students construct and manage identity in the context of pharmaceutical use	n = 16<br><br>8W & 8M<br><br>19-22Y.O.	Formal diagnosis n = 16	Semi-structured interview	Key symbol and theme analysis (Emerson, Fretz, and Shaw 1995)	All participants have been prescribed stimulants at some point in their lives.<br><br>Active prescriptions for medication n = 14	Behaviour modification n = 1<br><br>ADD Coach n = 1	However, while medicine may enable students to manage academic performance and take control of "disordered bodies," many remain uneasy about the extent to which they feel controlled by a drug. In the context of medical ambivalence, ADHD students engage in reflexive identity management and strategic pharmaceutical use to achieve some semblance of self-control and self-preservation during their college years. As their college education comes to a close, many prepare to return to what they construct as their "authentic," nonmedicated selves as they enter the work world.
Lyhne	2021	Denmark	Community mental health center	To examine perceived aspects of importance among young adults with ADHD to participate and engage in occupational activities, and to explain how	n = 8<br><br>4W & 4M<br><br>19-30Y.O.	Formal diagnosis n = 8	Semi-structured interview	Qualitative study with inductive content analysis methodology	Not reported	Individual Placement Support n = 8	Four categories emerged from the analysed interviews: (1) Being involved in an occupational environment fulfils a need for social contact, (2) Occupational activities must be clear and within interest (3) Self-confidence and daily routines are prerequisites for occupational participation (4) Having a lifeline providing continuous support is important.

				support from occupational specialists can assist them to deal with executive impairments							
Meaux	2009	United States	University	To determine factors that help, as well as hinder, college students with ADHD as they learn to cope with everyday challenges of life and academics once they leave the structure and support of their parents' home	n = 15<br><br>6W & 9M<br><br>recruitment range 18-21Y.O.	Formal diagnosis n = 15	Semi-structured interview	Qualitative descriptive methodology with thematic analysis	Using medication = 10<br><br>Regular use n = 3<br><br>Occasional use n = 7	Support from University Disability Support Services n = 6	Identification of three global themes: gaining insight about ADHD, managing life and utilizing sources of support. Each global theme contains factors that hinder, as well as factors that help the college student with ADHD.
Oster	2020	Sweden	Child and adolescent psychiatric outpatient units	Explore how adolescents with ADHD perceive and experience stress (and stressors) using a qualitative approach	n = 20<br><br>12W & 8M<br><br>15-18Y.O.	Formal diagnosis n = 20	Semi-structured interview	Qualitative study with qualitative content analysis	Using medication n = 16	Structured skills training group based on dialectical behavioral therapy n = 20	Stress and ADHD, as well as stress, anxiety and ill-health, were described as closely intertwined. The result is presented in four categories: stress is often present, triggers of stress, stress affects daily life, and stress can be handled and prevented. A relation was found between stress and feelings of helplessness, ill-health and anxiety. Stress was viewed as being out of proportion with reality and was driven by such factors as ADHD symptoms, school demands, unpredictable situations and relational problems. Several negative consequences of stress were reported, including postponing schoolwork and the tendency to

											give up. Some participants also reported performing better when stressed. Accepting help from others, practicing acceptance, settling down and controlling oneself, and planning in advance were seen as helpful stress managing techniques.
Perry	2006	United States	Colleges	Explore how undergraduate students diagnosed with AD/HD remain in college	n = 10<br><br>3W & 7M<br><br>18-24Y.O.	Formal diagnosis n = 10	Semi-structured interview	Qualitative research design with a grounded theory approach to analysis	Number of participants taking medication not explicitly stated.	Support from University Disability Support Services n = 10<br><br>Some used dietary control	The findings included themes related to attitudes about their diagnosis, adapting to college, and recommendations for students, college personnel, and parents.
Rasmussen	2024	Norway	Child and adolescent psychiatric service	To gain increased knowledge of emerging adults' experience of living with ADHD in the transition from adolescence to adulthood.	n = 7<br><br>2W & 5M<br><br>21-27Y.O.	Formal diagnosis n = 7	Semi-structured interview	Qualitative retrospective design with Malterud's systematic text condensation for analysis.	Experience of medication use n = 7	Psychotherapy for emotional regulation n = 1	Four crosscutting themes were identified from our analysis: (1) low level of knowledge about ADHD and treatment options; (2) barriers to seeking and accessing help; (3) developing self-help strategies; and (4) a preference to discontinued medication use.
Schaefer	2017	United States	University	Explore the medication self-management experiences of adolescents with attention-deficit/hyperactivity disorder (ADHD) during their transition to young adulthood in college	n = 10<br><br>3W & 7M<br><br>18.5-19.4Y.O.	Formal diagnosis n = 10	Semi-structured interview	Exploratory study with directed content analysis	Using medication n = 10	Academic accommodations n = 4	Five themes emerged from interviews: (1) transitions to independence are often abrupt, and many adolescents lack critical self-management skills; (2) volitional nonadherence is high due to inaccurate disease beliefs, perceived academic demands, and medication side effects; (3) poor self-management negatively impacts school performance; (4) peer pressure to share medication affects social functioning and adherence; and (5) social support is greatly needed.

Sibley	2018	United States	University clinic	To identify pre- and post-transition factors that influence post-secondary success for students with ADHD	n = 13<br><br>5W & 8M<br><br>18-21Y.O.	Formal diagnosis n = 13	Constructivist interviewing	Qualitative study with grounded theory framework for analysis	50.0% were currently receiving stimulant medication	No overarching description of non-pharmacological therapy use; individual findings note that some participants consulted mentors, therapists, in-patient mental health treatment, and university disability services	Informants offered their perspectives of what factors were critical to post-secondary success and how (i.e., by what mechanisms) these factors influence young adult functioning. Twenty macro themes were identified under three priori categories: motivational, skills, and environmental factors. Two macro themes outside of these categories were identified post hoc. Motivation and self-control difficulties were identified as the most commonly impairing deficits. Factors that enhanced and undermined these deficits were identified by parents and young adults.
Stamp	2014	United States	College	Examine the perceptions of college students with ADHD who were unable to pass an adequate number of classes, access appropriate sources of support, and/or advocate for themselves effectively in their first attempt at college	n = 12<br><br>4W & 8M<br><br>20-27Y.O.	Formal diagnosis n = 12	Semi-structured interview	Inductive analysis process	Consistent medication use n = 9<br><br>Discontinued use n = 3	No overarching description of non-pharmacological therapy use; individual findings note that some participants consulted university disability services	During semi-structured interviews, students were asked to share their perceptions of (1) the impact of ADHD on their experiences interacting with others and advocating for themselves in educational, work, and social settings; (2) how other people in society view ADHD and methods the students had used to cope with this disorder; and (3) interventions that had helped or hindered their efforts to adjust to the demands of college and advocate for themselves effectively. Their comments suggested several areas of difficulty including shame, avoidance to cope with distress, lack of understanding regarding the impact of ADHD, and poor awareness of available support for related difficulties
Tov	2022	Israel	Academic institute	Examine coping strategies adopted by overweight	n = 30<br><br>W & M not reported	Formal diagnosis n = 30	Semi-structured interview	Descriptive qualitative phenomenological	Not reported	No overarching description of non-pharmacological therapy use;	The study yielded two main themes. The first is struggling with disappointments and negative feelings in the past, and the second is the

				adults with ADHD to promote healthy behaviors and weight-control management.	24-46Y.O.			study with thematic content analysis		individual findings note that some participants engaged in weight-loss groups	reciprocity between weight management and coping skills strategies. The second theme includes three subthemes—cognitive strategies, behavioral strategies, and emotional strategies.
Weisner	2018	United States	Universities, a childrens hospital, university medical centre	Provide rich information on beliefs and expectations regarding ADHD, life's turning points, medication use, and substance use (SU)	n = 125<br><br>30W & 95M<br><br>23.3-25.5Y.O.	Formal diagnosis n = 125	Interview based on Ecocultural Family Interview	Qualitative coding and indexing	Number of participants taking medication not explicitly stated.	Not reported	ADHD youth more often desisted from SU because of seeing others going down wrong paths due to SU. Narratives revealed very diverse accounts and explanations for SU-ADHD influences.
Wiener	2016	Canada	N.R.	To gain rich, detailed information that could aid in service delivery to this population; without a better understanding of the school experiences of adolescents with ADHD, energy and resources may be wasted on strategies that have not been substantiated by research or are not	n = 12<br><br>3W & 9M<br><br>14-16Y.O.	Formal diagnosis n = 12	Semi-structured clinical interview	Qualitative study with modified grounded theory methodology and analysis	Regular medication use n = 8	No overarching description of non-pharmacological therapy use; individual findings note that some participants had Individualised Education Plans	Three themes emerged: (a) support for a performance deficit, (b) academic and social engagement, and (c) moving from dependence to independence. What is most striking is the low level of agency students demonstrated; that is, rather than acting with purpose on their environments, they seemed to react to things that happened to them.

				embraced by those for whom they are intended							
